# Land Use Compounds Habitat Losses under Projected Climate Change in a Threatened California Ecosystem

**DOI:** 10.1371/journal.pone.0086487

**Published:** 2014-01-21

**Authors:** Erin Coulter Riordan, Philip W. Rundel

**Affiliations:** Department of Ecology and Evolutionary Biology, University of California Los Angeles, Los Angeles, California, United States of America; DOE Pacific Northwest National Laboratory, United States of America

## Abstract

Given the rapidly growing human population in mediterranean-climate systems, land use may pose a more immediate threat to biodiversity than climate change this century, yet few studies address the relative future impacts of both drivers. We assess spatial and temporal patterns of projected 21^st^ century land use and climate change on California sage scrub (CSS), a plant association of considerable diversity and threatened status in the mediterranean-climate California Floristic Province. Using a species distribution modeling approach combined with spatially-explicit land use projections, we model habitat loss for 20 dominant shrub species under unlimited and no dispersal scenarios at two time intervals (early and late century) in two ecoregions in California (Central Coast and South Coast). Overall, projected climate change impacts were highly variable across CSS species and heavily dependent on dispersal assumptions. Projected anthropogenic land use drove greater relative habitat losses compared to projected climate change in many species. This pattern was only significant under assumptions of unlimited dispersal, however, where considerable climate-driven habitat gains offset some concurrent climate-driven habitat losses. Additionally, some of the habitat gained with projected climate change overlapped with projected land use. Most species showed potential northern habitat expansion and southern habitat contraction due to projected climate change, resulting in sharply contrasting patterns of impact between Central and South Coast Ecoregions. In the Central Coast, dispersal could play an important role moderating losses from both climate change and land use. In contrast, high geographic overlap in habitat losses driven by projected climate change and projected land use in the South Coast underscores the potential for compounding negative impacts of both drivers. Limiting habitat conversion may be a broadly beneficial strategy under climate change. We emphasize the importance of addressing both drivers in conservation and resource management planning.

## Introduction

The combined impacts of climate change and land use are expected to drive unprecedented rates of environmental change and biodiversity loss this century. Climate is a major driver of species distributions and rising temperatures over the last 100 years have already resulted in significant shifts in species ranges worldwide [1], [2]. With mean global surface temperatures expected to rise as high as 4°C by 2100 [3], the persistence of biodiversity may be contingent upon the ability of species to track suitable climatic conditions [4], [5]. Considerable attention has focused on predicting potential habitat losses and range shifts of individual species under 21^st^ century climate change, and subsequently forecasting species losses [6], [7] yet continued human land use poses a significant, and possibly more immediate threat to species persistence this century [8] through habitat conversion and degradation. The disconnect between relatively high resolution downscaled climate projections and the typically coarse resolution treatment of land use-land cover change projections, has contributed to the exclusion of future land use from assessments of impacts of future environmental change on species and ecosystems, which require local to regional scale analyses [9]. Only recently have studies begun to incorporate projections of both land use and climate change [10], [11].

Addressing projected land use in climate change assessments is particularly important for mediterranean-climate regions, where multiple drivers of environmental change are projected to cause some of the highest proportional biodiversity losses worldwide by 2100, chief among which is land use [8]. Harboring nearly 20% of the world's vascular plant species [12], these regions are global biodiversity hotspots [13]. Their dense, rapidly expanding human populations pose a significant threat to biodiversity, as evidenced by increasing numbers of species of conservation concern with growing human population density over the last decade [14]. Projected 21^st^ century climate change poses an additional threat, potentially driving dramatic range shifts and species losses across the biome [15]–[18], which may be further exasperated by high levels of human land use [19]. Thus, assessing the relative future impacts of both drivers is critical to prioritize conservation planning and effectively protect biodiversity in mediterranean-climate regions under conditions of environmental change.

Focusing on California sage scrub (CSS), a plant association of considerable diversity, endemism, and threatened status in the mediterranean-climate California Floristic Province, we investigate the relative impacts of projected 21^st^ century land use and climate change on CSS habitat suitability and richness. California has the greatest urbanization and population growth of all five mediterranean regions [14]. Nearly 25% of the state's >6,500 native plant taxa have a rare, threatened, or endangered status on federal and/or state agency lists, largely as a result of anthropogenic impacts including habitat degradation and destruction from land use [20]. Furthermore, California's current 37.5 million population is expected to grow to between 43.8 and 147.7 million by 2100 [21]. Substantial shifts in California's climate also pose a considerable threat to the state's biodiversity and ecosystems. General circulation models (GCMs) project an increase in annual mean temperature of 1.35°C to 5.8°C statewide by the end of the century [22], which could drive dramatic range losses for as many as two-thirds of the endemic species of California that comprise over 25% of the state's flora [16].

Within California, CSS coincides with areas of high human impact, occurring primarily in coastal and semiarid interior regions of southern California but also in scattered patches along the central California coast [23]–[25]. The coastal counties of southern California containing CSS (Ventura, Los Angeles, Orange, and San Diego) also house nearly half (45%) of the state's population, yet only account for seven percent of the state's total land area [26]. Land development combined with habitat conversion to annual grasses driven by anthropogenic practices have reduced CSS to as little as 10% of its original extent in the state [25], [27]–[30], resulting in a large number of associated threatened and sensitive species [31], [32]. High land values and development pressure in the region continue to make the conservation of CSS challenging [33]. With rapidly expanding human population in southern California, land use change poses an immediate threat to CSS [34]. In addition, climate change is projected to drive the contraction and replacement of mediterranean-climate conditions with warmer and drier conditions along coastal areas of southern California and northwestern Baja California by 2100 [19], [35], areas of high CSS diversity and endemism [23]–[25], [29]. This could cause species losses and changes in CSS diversity patterns as individual species shift in distribution in response to the expansion and contraction of mediterranean climates [36], [37]. Furthermore, multiple drivers of environmental change could compound CSS habitat loss [11], [38].

Successful CSS conservation this century will likely hinge upon prioritizing management efforts based on the relative impacts of both projected future land use and climate change. Using a species distribution modeling (SDM) framework, we investigate the relative threat of habitat loss from 21^st^ century projected land use and climate change for 20 CSS species. Species distribution models, which define a species range with respect to environmental variables (e.g., climate), provide a means to forecast projected climate change impacts on species and diversity [39]–[41]. We assess the extent to which habitat loss driven by future land use versus climate change will jeopardize key shrub species and alter species richness patterns. To test if the degree of threat posed by land use and climate change will vary temporally and spatially across CSS, we compare habitat loss impacts at two time intervals (early and late century) in two ecoregions in California (Central Coast and South Coast).

## Materials and Methods

### Study system and area

California sage scrub is a unique plant association characterized by dominant drought-deciduous shrubs (e.g., *Salvia*) with variable contributions of succulent and evergreen species and a diverse, herbaceous understory. It is primarily distributed in various community compositions along the coast in southern California, USA and northwestern Baja California, Mexico, with scattered patches along the central California coast and the semi-arid interior of southern California [23]–[25], [42]. The southern limit of CSS near El Rosario in Baja California (∼30°N) coincides with the southern extent of mediterranean-type climate in North America and the transition to more arid, desert conditions [42]. Within California, CSS is a high conservation priority, providing habitat for over 100 plant and animal species currently considered threatened, endangered, or of special conservation concern [31], [32].

At a regional scale, climate strongly influences distributional patterns of CSS species and floristic associations [25], [28], [43]–[45]. Initiating new growth after fall rains and growing through the coldest winter months, CSS species are typically limited to lower elevation areas with mild, wet winters and are negatively correlated with minimum winter temperatures [28]. In coastal southern California, CSS is distributed along a gradient of decreasing annual precipitation ranging from 450 to 250 mm [25]. Evapotranspirative stress during summer months, which is related to maximum summer temperature, also appears to be a major factor influencing species distributions [44]. Regionally, CSS occurs across a variety of soils that range broadly in fertility [25], [46]. Only at local scales, do topography and soil typically become important in influencing CSS floristic variability [47].

**Figure 1:**
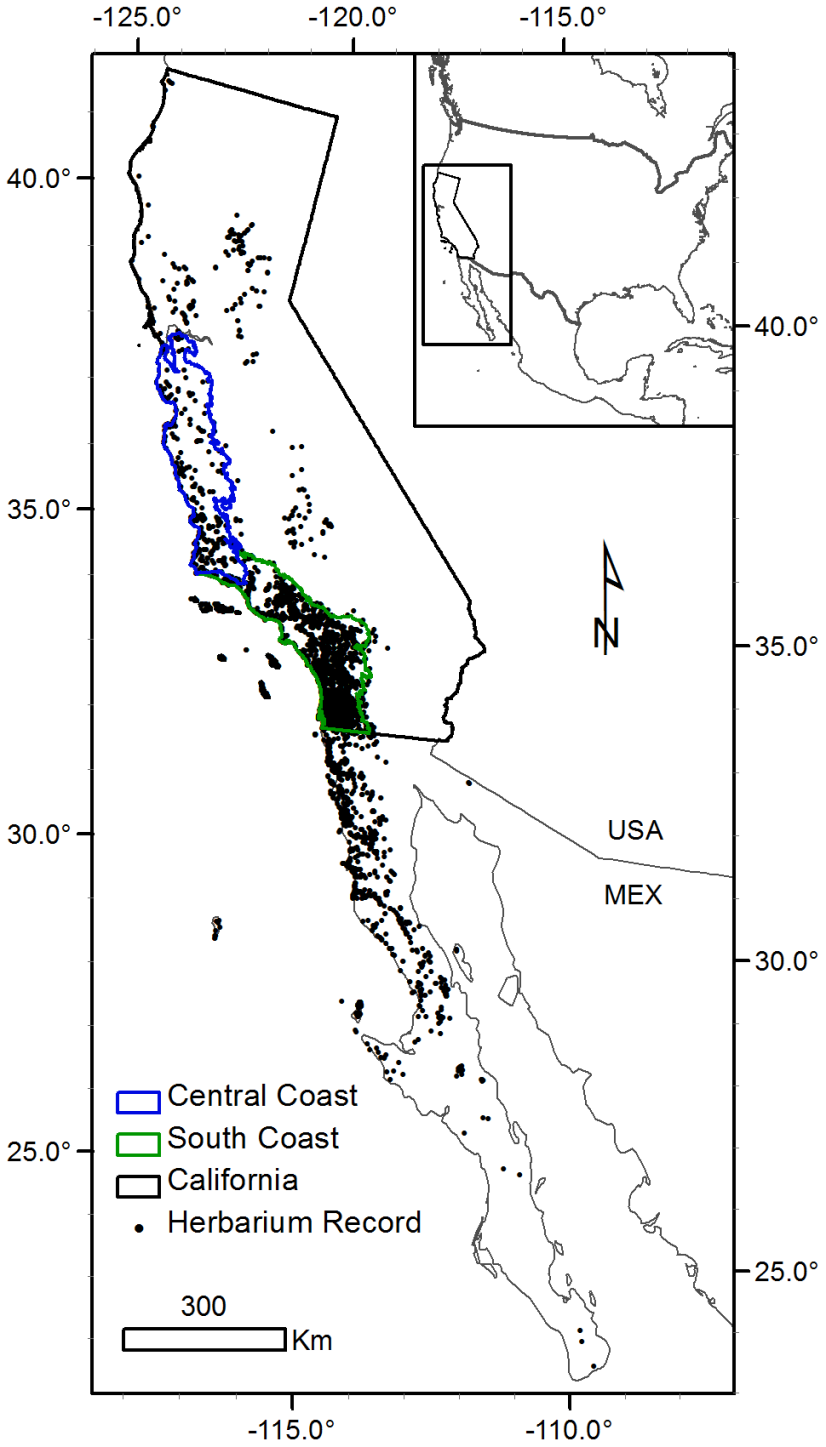
Study Area and Species Localities. Study area and herbarium record locations for the 20 modeled CSS shrub species. Habitat suitability models were created from all herbarium localities, analyses of habitat and land use change were restricted to within California.

We focused our analysis of the relative future impacts of land use and climate change on individual CSS species in California, where we have spatially explicit projections for both drivers of future habitat loss. We also analyzed geographic patterns of projected land use change and climate change impact on modeled CSS shrub species richness in two California ecoregions most critical to CSS: the Central Coast Ecoregion and South Coast Ecoregion, defined as “Central Western California” and “Southwestern California,” respectively, in *The Jepson Manual* [48] (Fig. 1). The Central Coast Ecoregion includes the San Francisco Bay Area at its northern limit, the central California coastline, the inner and other South Coast Ranges, and is bounded by the Santa Ynez Mountains to the south where it borders the South Coast Ecoregion. The South Coast Ecoregion includes the southern California coastline from Point Conception to the U.S.-Mexico border, the Transverse Ranges, and the Peninsular Ranges.

### Habitat suitability models

We used a SDM approach to model current and future climatically suitable habitat for 20 dominant CSS shrub species (Table 1). Such climate-based models have been used previously to identify regional patterns of habitat suitability for dominant shrub species and floristic groups in CSS [45]. While edaphic and topographic factors can be important drivers of CSS distribution at local scales (e.g., 50 meters) [47], this is beyond the scope of our analyses, which focus on regional patterns of projected land use and climate change impact on CSS. We modeled current species-climate relationships from herbarium record localities and climate data using Maxent (version 3.3.3) [49], a maximum-entropy modeling algorithm. We chose Maxent for its high performance with spatially biased presence-only data, such as our herbarium record localities [49]–[51]. The ability of Maxent to calculate probability distributions based on incomplete information is particularly useful for modeling CSS habitat suitability, where current CSS fragmentation patterns may reflect anthropogenic absences rather than environmental limits.

**Table 1:** List of California sage scrub species.

Taxon name	Family	Growth form	N	Mean test AUC (min–max)
*Acmispon glaber*	Fabaceae	Drought-deciduous subshrub	603	0.927 (0.905–0.942)
*Artemisia californica*	Asteraceae	Drought-deciduous shrub	242	0.957 (0.934–0.976)
*Bahiopsis laciniata*	Asteraceae	Drought-deciduous shrub	176	0.968 (0.925–0.986)
*Cneoridium dumosum*	Rutaceae	Evergreen shrub	117	0.974 (0.957–0.989)
*Encelia californica*	Asteraceae	Drought-deciduous shrub	204	0.969 (0.955–0.979)
*Ericameria ericoides*	Asteraceae	Evergreen shrub	85	0.991 (0.985–0.995)
*Eriogonum fasciculatum* (coastal vars.)	Polygonaceae	Evergreen shrub	665	0.923 (0.913–0.945)
*Hazardia squarrosa*	Asteraceae	Evergreen shrub	223	0.960 (0.923–0.967)
*Hesperoyucca whipplei*	Agavaceae	Evergreen shrub (rosette)	226	0.933 (0.880–0.952)
*Isocoma menziesii*	Asteraceae	Evergreen shrub	313	0.958 (0.948–0.962)
*Malosma laurina*	Anacardiaceae	Evergreen shrub	218	0.964 (0.949–0.975)
*Mimulus aurantiacus*	Phrymaceae	Drought-deciduous shrub	793	0.904 (0.890–0.922)
*Mirabilis laevis var. crassifolia*	Nyctaginaceae	Drought-deciduous subshrub	289	0.944 (0.922–0.952)
*Opuntia littoralis*	Cactaceae	Succulent shrub	79	0.978 (0.934–0.986)
*Rhus integrifolia*	Anacardiaceae	Evergreen shrub	196	0.977 (0.971–0.981)
*Ribes speciosum*	Grossulariaceae	Drought-deciduous shrub	138	0.977 (0.968–0.984)
*Salvia apiana*	Lamiaceae	Drought-deciduous shrub	305	0.948 (0.939–0.967)
*Salvia leucophylla*	Lamiaceae	Drought-deciduous shrub	60	0.982 (0.923–0.993)
*Salvia mellifera*	Lamiaceae	Drought-deciduous shrub	325	0.965 (0.946–0.972)
*Xylococcus bicolor*	Ericaceae	Evergreen shrub	189	0.977 (0.963–0.986)

Number of herbarium record localities (N) and overall model performance measured as the mean test area under the receiver operating characteristic curve (AUC) score (min–max). Taxonomy follows the second edition *The Jepson Manual: Vascular Plants of California* [48].

We obtained herbarium record localities collected from 1950 to present from the following herbarium databases: the Consortium of California Herbaria (CCH; http://ucjeps.berkeley.edu/consortium), the Southwest Environmental Information Network (SEINet; http://swbiodiversity.org/seinet), the Global Biodiversity Informatics Facility (GBIF; http://www.gbif.org), Baja Flora of the San Diego Natural History Museum (http://www.sdnhm.org), and the Red Mundial de Información sobre Biodiversidad (REMIB; http://www.conabio.gob.mx/remib/doctos/remib_esp.html) (Fig. 1). Prior to modeling, we mapped all records to identify and exclude cultivated plants, errors in georeferencing, obvious misidentifications, and duplicate collections.

We obtained current contemporary climate data from Worldclim (http://www.worldclim.org), a set of 19 bioclimatic variables derived from weather station monthly mean temperature and precipitation data collected from 1950 to 2000 and interpolated to 30-arcsec (ca. 1 km) resolution [52]. Finer resolution climate data exist for California [53], but exclude the warmer and drier climatic limit of many CSS species in Baja California. Furthermore, fine scale climate datasets (<1 km resolution) may not be appropriate for modeling species distributions from herbarium records, as uncertainties in georeferenced locations may limit the accuracy of locality data to coarser resolutions.

We narrowed the 19 bioclimatic variables to those thought to drive regional patterns of CSS distribution (continentality, temperature extremes, water availability, and seasonality in both temperature and precipitation). For highly correlated variable pairs (Pearson correlation coefficient | *r* |>0.80), we retained the variable with the highest contribution to model performance. Thus, our final seven bioclimatic variables minimized correlations among variables, maximized contribution to model predictions, and represented annual climate trends, seasonality, and extremes relevant to CSS distribution: annual mean temperature (BIO1), temperature seasonality (BIO4), maximum temperature of the warmest month (BIO5), minimum temperature of the coldest month (BIO6), precipitation seasonality (BIO15), precipitation of the warmest quarter (BIO18) and precipitation of the coldest quarter (BIO19).

We selected future climate scenarios representing two possible trajectories of climate change in California under the Intergovernmental Panel on Climate Change Special Report on Emission Scenarios (IPCC-SRES) A1B storyline: (1) a warmer wetter future (CCCMA CGC 3.1) and (2) a warmer drier future (NCAR CCSM 3.0). We obtained 30-arcsec (ca. 1 km) spatial resolution future climate data downscaled from GCM outputs of the IPCC Fourth Assessment Report [3] by the climate change program of the International Center for Tropical Agriculture (CIAT; http://www.ccafs-climate.org/data). We represent 21^st^ century climate at two time steps created from separate 30 year averages, one mid-century (2050s: 2040–2069) and one late-century (2080s: 2070–2100).

We modeled current climate-species relationships in Maxent using all herbarium records to train each species model, then projected these relationships onto future climate layers at two time periods, 2050s and 2080s. For each species model, a constant set of 10,000 background pixels selected randomly over the study area were used as “pseudo absences,” the maximum set of iterations was 500, the convergence threshold was set to 10^−5^, and regularization was set to “auto” allowing Maxent to set the amount of regularization automatically based on our locality and environmental data [54]. We used 10-fold cross-validation to replicate model runs and estimate evaluation statistics for each species. We measured overall model performance using the area under the receiving operator characteristics curve (AUC), which ranges from 0.5 (random prediction) to 1 (maximum prediction) and can be interpreted as the ability of model predictions to discriminate presence sites from random background [49].

We created binary current and future habitat maps (0 = unsuitable, 1 = suitable) from Maxent's logistic output using the maximum sensitivity plus specificity threshold which provides among the most accurate predictions for presence-only data [55], [56]. We calculated habitat loss and gain from climate change using two different future dispersal scenarios, a best-case, unlimited dispersal scenario where species can colonize any future suitable habitat, and a worst-case, no dispersal scenario where species cannot disperse to future suitable habitat falling outside of modeled currently suitable habitat. We estimated future CSS species richness by overlaying individual species habitat maps under each climate change and dispersal scenario.

### Current and future land use-land cover

We used current (2000) and projected future (2050 and 2080) land use-land cover maps (Fig. S1) consistent with the IPCC-SRES A1B scenario and developed by the United States Geological Survey (USGS) [9]. These spatially explicit, high resolution (250 m) maps were constructed for 84 ecoregions across the conterminous United States using the FORecasting SCEnarios of future land-use (FORE-SCE) model [57] and integrate both “top-down” drivers of land cover/land use such as demographic change, and local-scale “bottom-up” drivers such as biophysical site conditions [9]. The A1B scenario we considered in our study represents one possible future storyline characterized by rapid economic growth, a global population that peaks in mid-century and declines thereafter, rapid technological innovation, balanced energy sources, and active management of resources [3]. Under this scenario, projected urban growth is high, particularly in coastal areas and near urban centers, large increases in biofuel and food production drive large expansions in agricultural lands, and increased fragmentation of natural land covers is projected in regions with well-developed infrastructure and abundant natural resources [9]. The USGS land use-land cover maps provide five categories of human land use: developed, representing areas of both intensive (e.g., urban and built-up environments, high density housing) and less intensive (e.g., low density housing, parks) uses, cultivated cropland, mechanically disturbed representing areas of forest harvest and clear-cut logging, mining, and hay/pasture. We examined each human land use category individually as well as combined into a single “anthropogenic land use” category. We resampled the 250 m resolution land use-land cover data to match the 1 km resolution of our climate data using the nearest neighbor method in ArcGIS 10.0 (ESRI, Redlands, CA, USA).

### Relative projected land use and climate change impacts

We overlaid habitat suitability maps with current and projected land use data to estimate the percent change in habitat under three scenarios, land use only, climate change only, and combined land use and climate change, and two time periods, early century (2000–2050) and late century (2050–2080). We assumed a complete loss of habitat in areas of anthropogenic land use (developed, mechanically disturbed, mining, cultivated croplands, and hay/pasture). All percentages of habitat change were relative to the amount of unconverted current climatically suitable habitat in 2000. In combined land use and climate change scenarios, we calculated the percent overlap in habitat lost or gained due to projected climate change and habitat lost due to projected land use. To investigate the potential impact of both drivers on CSS diversity, we overlaid projected land use change maps with maps of species richness change due to projected climate change. We limited our comparisons of projected land use and climate change impact on individual CSS species to California and CSS species richness to Central Coast and South Coast California Ecoregions. Maxent habitat suitability models, however, were based upon the full range of location data (California and Baja California, Mexico), as using spatially truncated locality data can lead to over-predicting range losses under future climate change [58]. All spatial analyses and model visualizations were performed in ArcMap 10.0 (ESRI, Redlands, CA, USA).

## Results

### Projected land use-land cover change

**Figure 2:**
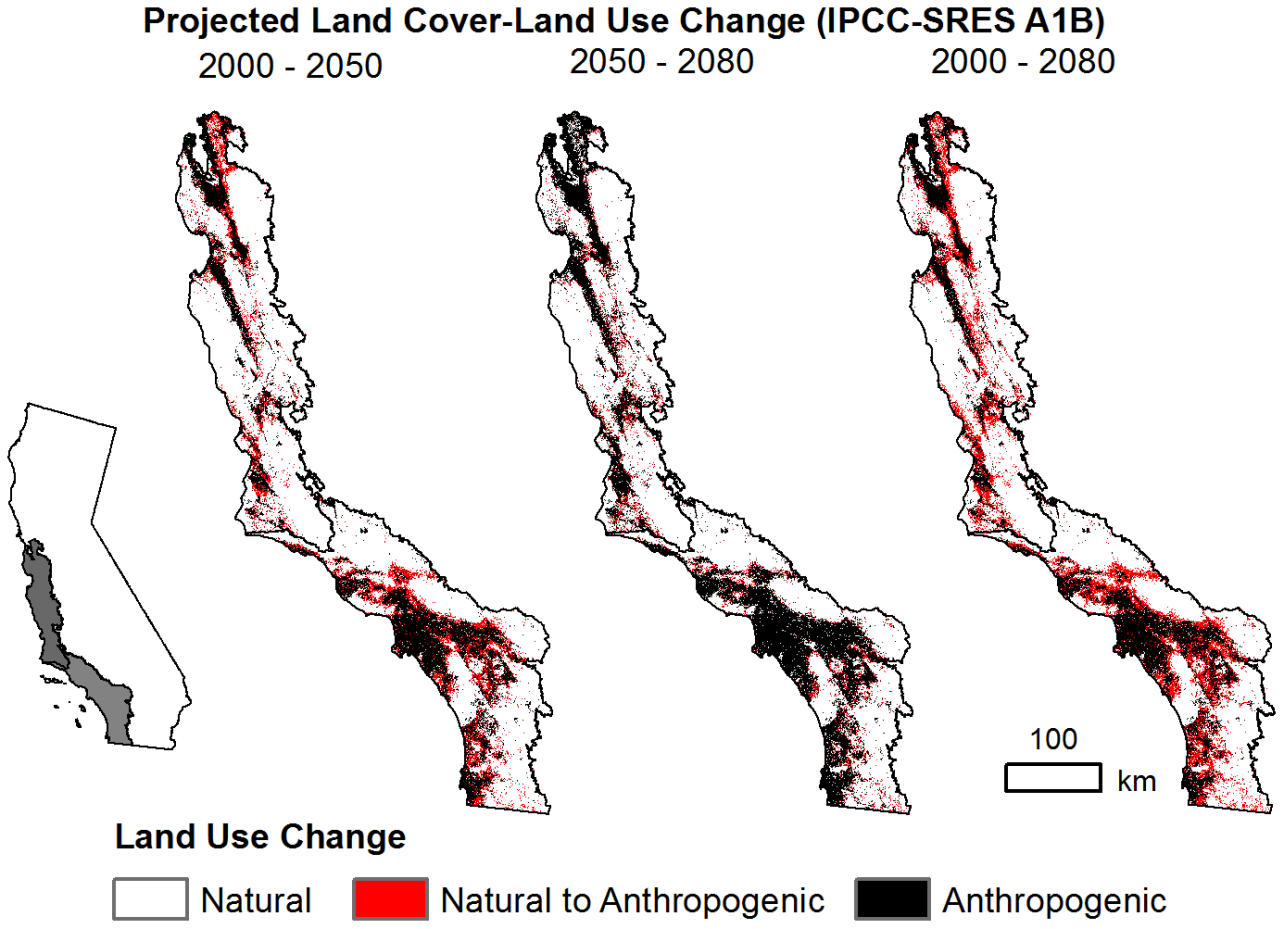
Projected Anthropogenic Land Use Change Maps. Projected 21^st^ century change in anthropogenic land use (2000–2050, 2050–2080, 2000–2080) under the IPCC-SRES A1B scenario. Anthropogenic uses include developed areas, cultivated crops, hay/pasture, mining, and mechanically disturbed (logged) land. Land use-land cover maps were resampled to 1 km resolution from the USGS LandCarbon 250 m resolution land use-land cover maps for the continental United States [9].

Already, anthropogenic surfaces cover 15.6% of the Central Coast and 24.3% of the South Coast (Table S1, Fig. 2). Under the A1B scenario considered in our study, USGS land use-land cover projections show increasing anthropogenic land uses converting natural land covers at rates of 81 km^2^yr^−1^ (Central Coast) and 117 km^2^yr^−1^ (South Coast) early century (2000–2050), slowing to 78 km^2^yr^−1^ and 75 km^2^yr^−1^ late century (2050–2080) (Table S1). By 2080, anthropogenic land uses may cover a total of 14,107 km^2^, or nearly 30%, of the Central Coast and 19,628 km^2^, just over 40%, of the South Coast (Fig. 2). Much of this conversion will be driven by increasing development concentrated near major metropolitan areas such as the San Francisco Bay Area, Los Angeles, and San Diego (Fig. S1). By 2080, development is projected to increase by 4,661 km^2^ (Central Coast) and 8,379 km^2^ (South Coast) (Table S1). Cultivated croplands are also projected to increase in the Central Coast, covering an additional 1,413 km^2^ by 2080. Unlike development, which is projected to slow late century in both ecoregions, the expansion of agricultural land uses (cultivated crops and hay/pasture) is projected to increase at a greater rate late century, though only in the Central Coast. In the South Coast, increasing agricultural land uses will be paired with intensive development of already existing agricultural lands, especially early century, leading to net losses by 2080 (Table 2, Table S1). Much of the projected increase in anthropogenic land uses in the Central and South Coast will be at the expense of grasslands and shrublands (Table 2). The high projected conversion of shrublands is particularly pertinent to CSS, which is included within the shrubland land cover. Projected shrubland decline is greatest in the South Coast with a 5,658 km^2^ (26.4%) loss from 2000 to 2080, 4,575 km^2^ of which will be due to development, the greatest type of projected land use-land cover change for the ecoregion. In the Central Coast, shrublands are projected to decline by 1,347 km^2^ (15.2%) from 2000 to 2080, with development, cultivated croplands, and hay/pasture driving similar degrees of conversion, 423–458 km^2^ (Table 2).

**Table 2:** Primary projected changes in land use-land cover for the Central Coast and South Coast California Ecoregions.

	2000–2050		2050–2080		2000–2080	
Land Cover	Area (km^2^)	% Ecoregion	Area (km^2^)	% Ecoregion	Area (km^2^)	% Ecoregion
**Central Coast**						
Grassland to developed	2047	4.1	716	1.4	2791	5.6
Grassland to cultivated cropland	786	1.6	762	1.5	1550	3.1
Cultivated cropland to developed	600	1.2	236	0.5	789	1.6
Grassland to hay/pasture	359	0.7	241	0.5	494	1.0
Hay/pasture to developed	285	0.6	128	0.3	478	1.0
Shrubland to cultivated cropland	275	0.6	228	0.5	458	0.9
Shrubland to developed	243	0.5	131	0.3	433	0.9
Shrubland to hay/pasture	222	0.4	225	0.5	423	0.9
**South Coast**						
Shrubland to developed	3181	6.7	1239	2.6	4575	9.6
Grassland to developed	1763	3.7	359	0.8	2128	4.5
Cultivated cropland to developed	811	1.7	330	0.7	1002	2.1
Shrubland to cultivated cropland	533	1.1	488	1.0	891	1.9
Hay/pasture to developed	382	0.8	110	0.2	464	1.0
Shrubland to hay/pasture	130	0.3	61	0.1	140	0.3
Grassland to cultivated cropland	75	0.2	50	0.1	122	0.3
Evergreen forest to developed	70	0.1	31	0.1	102	0.2

The change in land use-land cover is shown as an area (km^2^) and as the percent of total land area in each ecoregion. Calculations are based on 1 km resampled USGS LandCarbon 250 m land use-land cover maps [9].

### Current land use impact on CSS habitat

Overall, Maxent models performed well with a median AUC score of 0.964 (range: 0.904–0.991) for the 20 shrub species in our study, suggesting climate is important in determining regional patterns of CSS habitat suitability (Table 1). Currently, a median 34.7% (range = 19.4–47.5%) of climatically suitable habitat of CSS species has already been converted to anthropogenic land uses (Table S2), with development driving the greatest loss in habitat (median 20.6%, range = 10.4–35.0%), followed by cultivated croplands (median 7.5%, range = 5.6–12.2%), and hay/pasture (median 3.9%, range = 2.1–5.2%). Six species have already lost over 40% of their climatically suitable habitat: *Xylococcus bicolor*, *Rhus integrifolia*, *Encelia californica*, *Opuntia littoralis*, *Bahiopsis laciniata*, and *Cneoridium dumosum* which has the greatest percentage of converted suitable habitat (47.5%). These species are distributed primarily in coastal southern California to northwestern Baja California and are most impacted by land development, which accounts for over 30% of current habitat losses in California. Widely distributed CSS shrub species ranging beyond coastal habitats in central and southern California (*Hesperoyucca whipplei, Mimulus aurantiacus, Eriogonum fasciculatum, Hazardia squarrosa, Acmispon glaber*) tend to have a lower degree of total habitat conversion and lower impact of development, which accounts for less than 15% of their current habitat losses (Table S2).

### Relative impacts of projected land use and climate change

#### Habitat loss

Under the A1B scenario we considered, anthropogenic land use alone is projected to drive a median 22.1% loss (range = 12.7–34.8%) of currently unconverted climatically suitable habitat for dominant CSS shrub species by 2050 and an additional median 9.1% loss (5.9–11.0%) from 2050 to 2080 (Table 3). Projected development poses the greatest threat to all species, accounting for a median 16.7% (8.9–30.4%) habitat loss early century (2000–2050), nearly 4 times greater than the combined threats of all other anthropogenic land uses during that period (Table 3). Late century (2050–2080) development poses a lesser but still notable threat, accounting for a median 5.3% (3.3–8.1) habitat loss, which is 1.8 times greater than the combined median losses of other anthropogenic land uses during that period. Habitat conversion to cultivated cropland and hay/pasture pose relatively low threats, driving median 3.2% (2.0–4.7%) and 2.2% (1.6–3.5%) losses early century and 1.2% (<0.1–0.6%) and 0.8% (0–0.7%) losses late century, respectively. Mining and logging poses negligible threats, accounting for less than 0.1% of total habitat losses. The species with the greatest projected habitat losses continue to be those distributed primarily in coastal southern California and northwestern Baja California, with six species projected to lose 40% or more of unconverted, climatically suitable habitat to anthropogenic land uses by 2080 (Table S2).

**Table 3:** Summary of loss of CSS species suitable habitat to projected anthropogenic land uses.

	Median (min–max) percent loss in CSS habitat		
Land use type	2000–2050	2050–2080	2000–2080
Development	16.7 (8.9–30.4)	5.3 (3.3–8.1)	22.7 (12.4–39.5)
Cultivated crops	3.2 (2–4.7)	2.2 (1.6–3.5)	4.9 (3.4–7.7)
Hay/pasture	1.2 (0.4–1.8)	0.8 (0.3–1.2)	1.9 (0.6–2.6)
Other	0.1 (<0.1–0.6)	<0.1 (0–0.7)	0.1 (0–0.8)
Total anthropogenic land use	22.1 (12.7–34.8)	9.1 (5.9–11)	31.2 (18.3–45.6)

“Other” land use category includes mining and mechanically disturbed (logging) categories. Table values are the median percent loss (min–max) of modeled current climatically suitable habitat that was unconverted in 2000.

**Figure 3:**
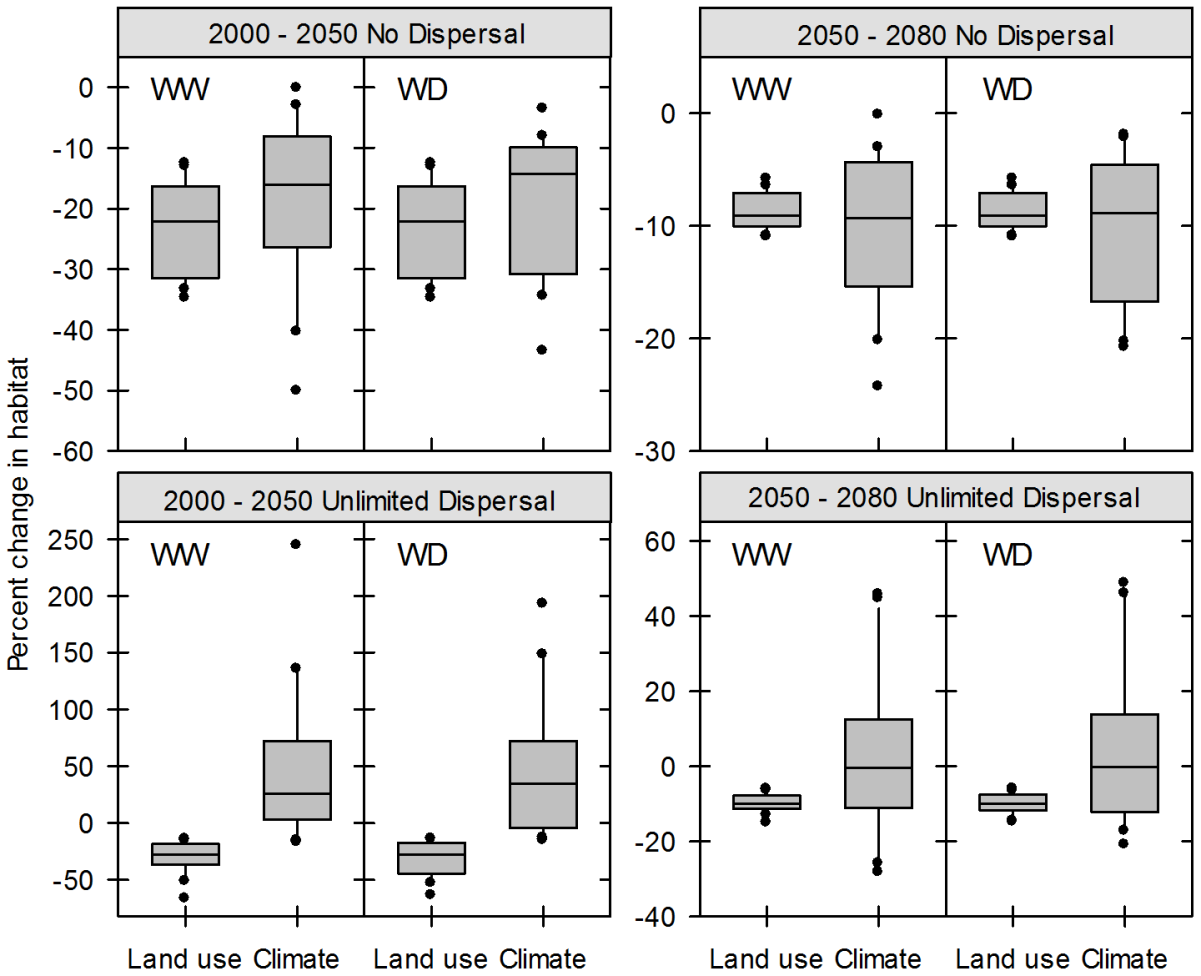
Relative Impacts of Projected Land Use and Climate Change on CSS Species Habitat. Boxplots showing the percent change in CSS species habitat due to projected land use and climate change under the A1B IPCC-SRES scenario. Climate scenarios are abbreviated as WW (warmer wetter CCCMA CGC 3.1) and WD (warmer drier NCAR CCSM 3.0). No dispersal and unlimited dispersal scenarios are shown separately.

In comparison, projected climate change has a much more variable impact on CSS species, potentially driving both losses and gains in climatically suitable habitat (Fig. 3). Overall, we find patterns of northern habitat expansion and southern habitat contraction with 21^st^ century climate change consistent across warmer wetter and warmer drier climate change trajectories. Assuming scenarios of no dispersal, where species cannot disperse into new climatically suitable habitat, early century climate change alone is projected to drive a median habitat loss of 16.0% (0.2–50.2%) under a warmer wetter future and 14.3% (3.6–43.6%) under a warmer drier future (Table 4). Projected losses increase by a median 9.3% (0.2–24.3%; warmer wetter) and 8.8% (2.0–20.8%; warmer drier) late century, resulting in median cumulative projected habitat losses of 24.6% (0.5–83.0%) and 24.5% (6.8–61.2%), respectively, by the end of the century (2080s). Ten species show cumulative projected climate-driven habitat losses of greater than 40% under at least one climate scenario (Table S3, Table S4). In contrast to the pattern of greater projected land use impact for more narrowly distributed south-coastal species, we find large habitat losses from projected climate change for both widely distributed (e.g., *E. fasciculatum*) and south-coastal species (e.g., *X. bicolor*).

**Table 4:** Summary of percent change in suitable CSS species habitat under 21^st^ century land use and climate change scenarios.

Scenario	GCM	Dispersal	Median (min to max) percent change in habitat		
			2000–2050	2050–2080	2000–2080
Land use only	None	N/A	−22.1 (−34.8 to −12.7)	−9.1 (−11 to −5.9)	−31.2 (−45.6 to −18.3)
Climate change only	WW	No	−16 (−50.2 to −0.2)	−9.3 (−24.3 to −0.2)	−24.6 (−83 to −0.5)
		Yes	25.7 (−17.4 to 243.9)	−0.6 (−28.4 to 45.6)	24.5 (−50 to 288.3)
	WD	No	−14.3 (−43.6 to −3.6)	−8.8 (−20.8 to −2)	−24.2 (−61.2 to −6.8)
		Yes	34.5 (−15.3 to 192.4)	−0.2 (−21.1 to 48.6)	35.4 (−32.6 to 240.1)
Climate change and land use	WW	No	−35.1 (−64.6 to −23)	−14.7 (−25.4 to −9.6)	−46.7 (−89.9 to −30.2)
		Yes	11.8 (−43.6 to 176.9)	−7 (−37.5 to 21.8)	1.4 (−62.8 to 193.9)
	WD	No	−37.8 (−51.4 to −26.3)	−16 (−24.2 to −10.2)	−54 (−75.4 to −35.3)
		Yes	9.8 (−25.4 to 130)	−9.4 (−26.1 to 23.3)	1.3 (−43.1 to 151.9)

Table values are the median percent change (min to max) of modeled current climatically suitable habitat that was unconverted in 2000. Climate scenarios (GCM) are abbreviated as WW (warmer wetter; CCCMA CGC 3.1) and WD (warmer drier; NCAR CCSM 3.0).

**Figure 4:**
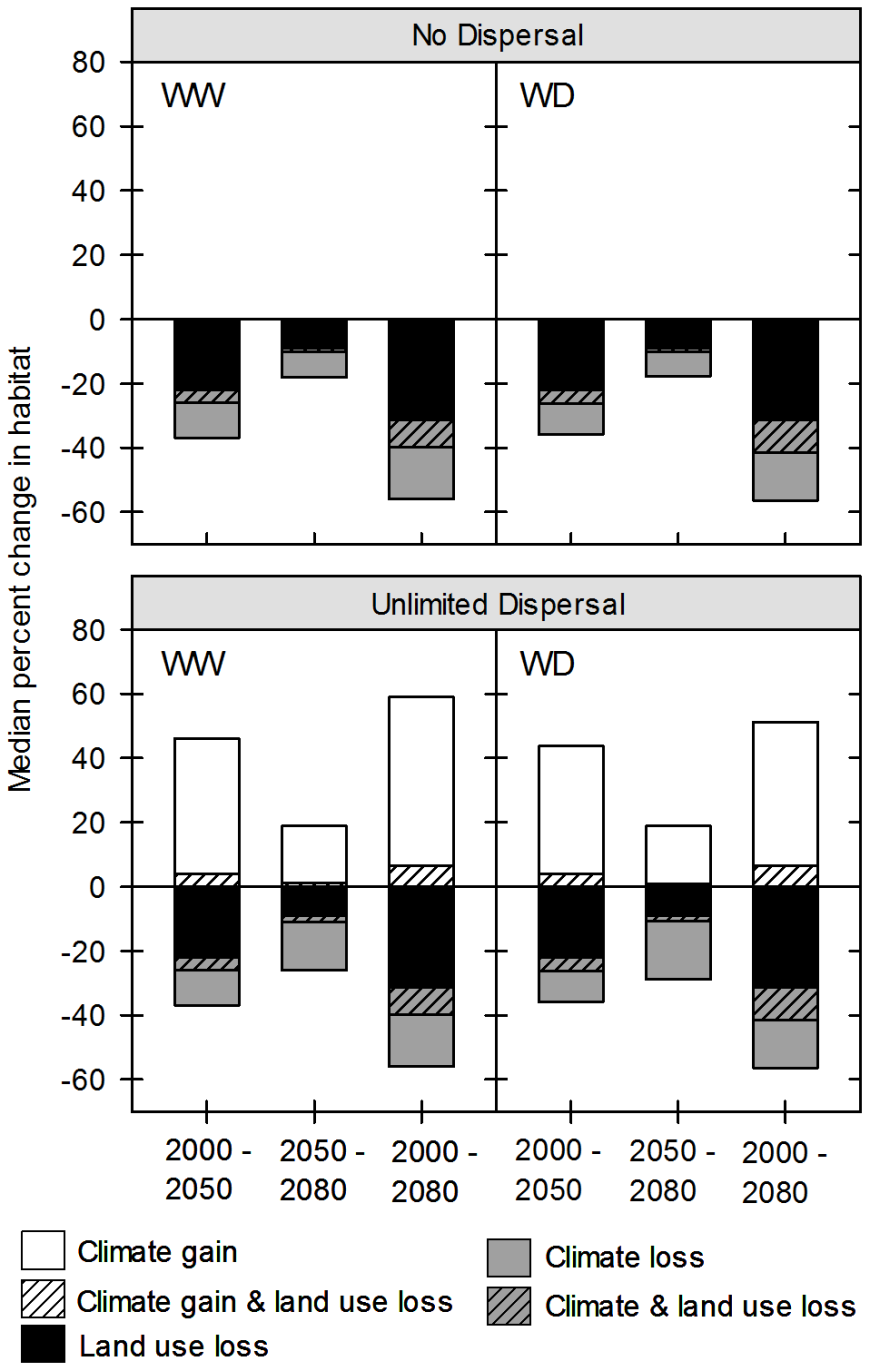
Median Percent Change in CSS Species Habitat from Projected Land Use and Climate Change. Plots show the median percent changes in species habitat due to projected land use and climate change, including percent overlap of impacts, under each climate and dispersal scenario. Climate scenarios are abbreviated as WW (warmer wetter CCCMA CGC 3.1) and WD (warmer drier NCAR CCSM 3.0).

When impacts due to projected land use are combined with those due to projected climate change under assumptions of no dispersal, overall habitat losses increase to a median 35.1% (23.0–64.6%, warmer wetter) and 37.8% (26.3–51.4%, warmer drier) by the 2050s (Table 4). Late century projected losses increase an additional median 14.7% (9.6–25.4%, warmer wetter) and 16.0% (10.2–24.2%, warmer wetter), leading to median cumulative losses of ∼50%. Only two species, *Artemisia californica* and *Ericameria ericoides*, show projected losses of less than 40% under at least one climate scenario. We also find notable spatial overlap in habitat loss from both drivers (Fig. 4), a median 26.9% (15.8–45.5%, warmer wetter) and 24.5% (13.4–41.5%, warmer drier) of climate-driven losses overlap with projected land use early century, and a median 12.9% (9.7–25.8%, warmer wetter) and 12.5% (6.6–22.5%, warmer drier) overlap late century.

Assuming unlimited dispersal where species can fully expand into all areas of new climatically suitable habitat, climate change is projected to drive habitat gains for many of CSS species we considered, offsetting some habitat losses (Table S3, Table S4). Projected climate change impacts, however, are highly variable across species (Fig. 3): four species show net habitat gains of 100–200% by the end of the century (2080s) under at least one climate scenario before accounting for projected land use, while nine species show projected net habitat losses under at least one climate scenario (Table S3, Table S4). Under a warmer wetter climate scenario we find a median net habitat gain of 25.7% (−17.4–243.9%) early century and a median net habitat loss of 0.6% (−28.4–45.6%) by late century (Table 4). Under a warmer drier climate scenario, we find a median net habitat gain of 34.5% (−15.3–192.4%) early century and a median net habitat loss of 0.2% (−21.1–48.6%) late century. While nearly all (18) of the CSS species we considered show projected net habitat gains from climate change under at least one climate scenario early century, this number drops to 12 species late century, with only six species showing projected net habitat gains under both climate scenarios (Table 5). Overall, by the end of the century, the majority of species still show a cumulative net increase in suitable habitat due to climate change alone, though the degree of climate change impact remains highly variable among species.

**Table 5:** Sensitivity of individual CSS species to projected land use (LU) and climate change (CC).

	Net CC gain (unlimited dispersal)				LU>CC (unlimited dispersal)				LU>CC (no dispersal)			
	2000–2050		2050–2080		2000–2050		2050–2080		2000–2050		2050–2080	
Species	WW	WD	WW	WD	WW	WD	WW	WD	WW	WD	WW	WD
*Artemisia californica*	X	X	X	X	X	X	X	X	X	X	X	X
*Encelia californica*	X	X	X	X	X	X	X	X	X	X	X	X
*Acmispon glaber*	X	X	X	X	X	X	X	X	X	X	X	X
*Malosma laurina*	X	X	X	X	X	X	X	X	X	X	X	X
*Opuntia littoralis*	X	X	X	X	X	X	X	X	X	X	X	X
*Rhus integrifolia*	X	X	X	X	X	X	X	X	X	X	X	X
*Isocoma menziesii*	X	X		X	X	X	X	X	X	X	X	X
*Ericameria ericoides*	X	X		X	X	X		X	X	X	X	X
*Bahiopsis laciniata*	X	X		X	X	X		X	X	X		X
*Mirabilis laevis var. crassifolia*	X	X	X		X	X	X	X	X	X		
*Salvia mellifera*	X	X	X		X	X	X	X	X	X		
*Ribes speciosum*	X	X			X	X	X	X	X	X		
*Salvia apiana*	X	X			X	X						
*Mimulus aurantiacus*	X		X		X	X	X					
*Cneoridium dumosum*		X			X	X	X	X	X	X		X
*Xylococcus bicolor*		X			X	X		X				
*Hesperoyucca whipplei*	X				X	X						
*Salvia leucophylla*	X				X	X	X				X	
*Eriogonum fasciculatum*					X	X						
*Hazardia squarrosa*					X	X						

Climate scenarios are abbreviated as WW (warmer wetter; CCCMA CGC 3.1) and WD (warmer drier; NCAR CCSM 3.0). Species with an “X” in the first four columns have projected net habitat gains due to climate change assuming unlimited dispersal. An “X” in the remaining columns indicates a greater threat of habitat loss due to projected land use relative to that due to projected climate change. Species sensitivities are broken into unlimited dispersal and no dispersal assumptions.

When impacts from projected land use are combined with those from projected climate change under assumptions of unlimited dispersal we find only a slight, cumulative net gain in species habitat by the end of the century, with median projected habitat increases of just 1% between 2000 and the 2080s under both climate scenarios (Table 4). This is due to projected habitat conversion to anthropogenic land uses in both the climatically stable portions of species ranges and areas of projected habitat gain from climate change (Fig. 4). Early century, a median 8.8% (2.3–32.7%, warmer wetter) and 9.1% (2.9–19.1%, warmer drier) of the habitat gained under climate change will be lost to projected anthropogenic land use. Similarly, a median 5.5% (0.8–0.11.6%, warmer wetter) and 4.4% (2.3–7.2%, warmer drier) of the habitat gained late century will be lost to projected anthropogenic land use.

When considered separately, the relative impacts of projected land use and climate do not differ significantly across CSS species during either early or late century under assumptions of no dispersal (all *P*>0.20; two-tailed Wilcoxon Signed Rank Test). While not statistically significant, we do find a pattern of increasing relative threat from climate change during the second part of the century. Early century, projected land use poses a greater threat than projected climate change for 13 species, decreasing to 9–10 species late century, depending upon climate change scenario (Table 5). Under assumptions of unlimited dispersal, habitat gains from projected climate change offset some of the habitat losses also due to climate change, resulting in a greater relative threat of projected land use for all 20 CSS species early century (Table 5). Similar to the no dispersal scenario, the threat posed by projected climate change begins to increase late century with 6–7 species showing greater losses in habitat due to projected climate change relative to losses due to projected land use. Overall, land use is projected to drive greater habitat losses relative to climate change both early and late century under assumptions of unlimited dispersal (all *P*<0.04; two-tailed, paired Wilcoxon Signed Rank test).

#### Species richness

**Figure 5:**
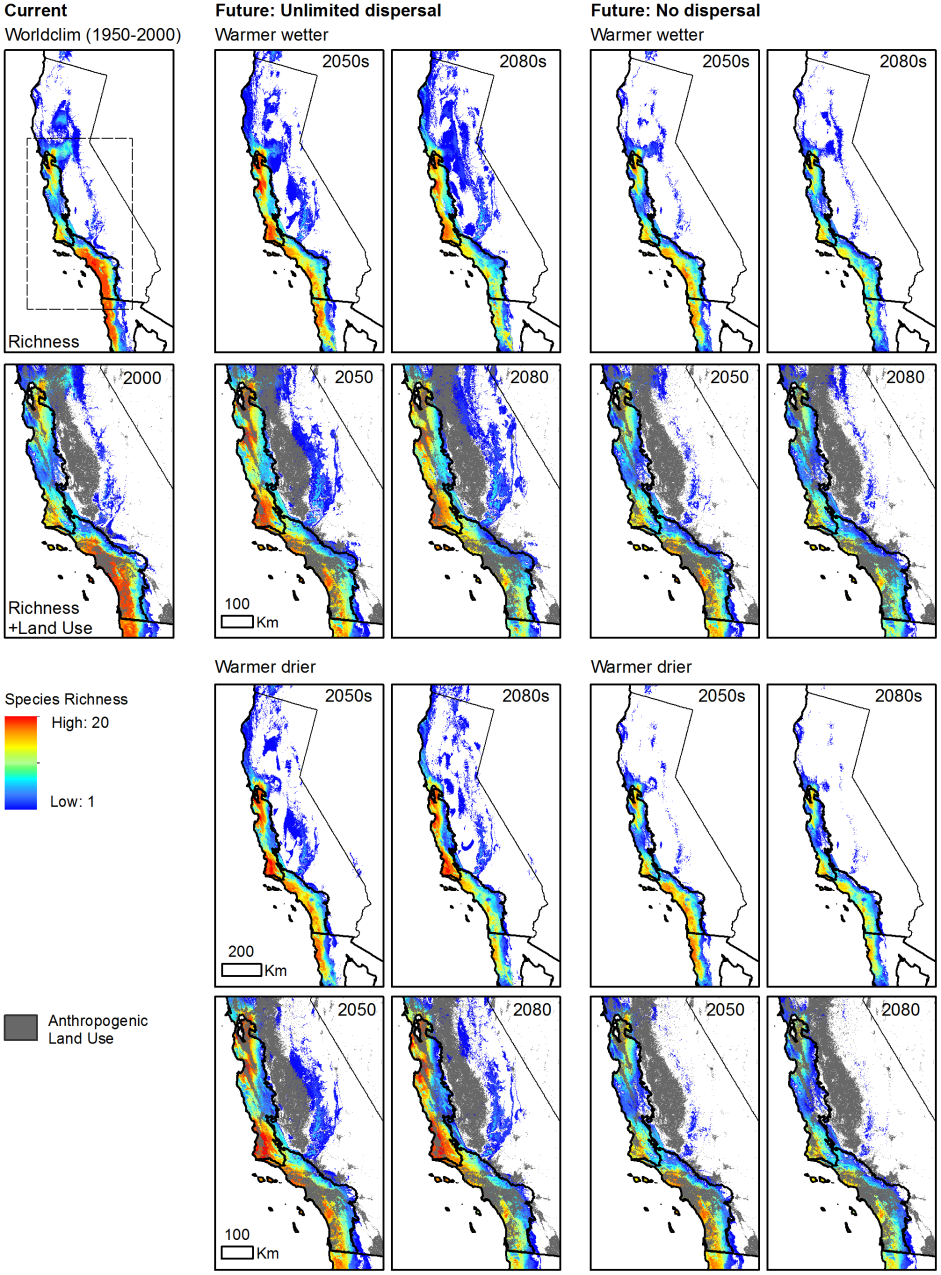
Projected Climate Change and Land Use Impacts on CSS Richness. Current (2000) and projected early century (2050s) and late century (2080s) CSS species richness under two climate change scenarios (top rows: warmer wetter CCCMA CGC 3.1, bottom rows: warmer drier NCAR CCSM 3.0) assuming unlimited dispersal and no dispersal. Anthropogenic land use is shown as gray overlays and includes developed, mining, mechanically disturbed (logging), cultivated croplands, and hay/pasture land uses.

Under the scenarios considered in our study, patterns of species richness under projected climate change varied geographically across ecoregions (Fig. 5). Currently, modeled CSS shrub species richness is centered in the South Coast Ecoregion in lowland, coastal areas. Under both future climate scenarios, we find considerable declines in modeled species richness due to climate change alone throughout much of the South Coast resulting from the southern contraction of climatically suitable habitat in many CSS species. Under best-case, unlimited dispersal scenarios, only about a quarter (23.3–28.7%) of the land area in the South Coast is projected to experience a net gain in richness due to climate change by the 2080s and 66.4–70.4% of the ecoregion will experience a net loss in richness (Table 6). Losses in species richness due to climate change alone will be greatest under assumptions of no dispersal, with >75% of the ecoregion losing richness. We find a much different outcome in the Central Coast Ecoregion, where northern habitat expansion due to projected climate change for many of our species, assuming unlimited dispersal, results in increased species richness by the end of the century. Assuming unlimited dispersal, over 70% of the Central Coast could experience an increase in species richness due to climate change by the end of the century, with only 12.9–21.8% of the ecoregion experiencing losses in richness (Table 6). Even assuming no dispersal, Central Coast losses in species richness due to climate change are less severe than the South Coast, covering just over half (51.8–55.8%) of the ecoregion by the end of the century.

**Table 6:** Percent of land area with projected loss and gain in CSS species richness due to climate change summarized by land use category.

			Land area (km^2^)		% Land area with richness loss		% Land area with richness gain	
Ecoregion	GCM	Land use	2050	2080	2050	2080	2050	2080
**Central Coast**	WW	Natural	37646	35324	1.8 (37.76)	11.3 (47.7)	93.3	82.2
		Natural to Anthropogenic	4124	6448	0.5 (35.0)	14.6 (59.7)	96.3	76.5
		Anthropogenic	7662	7667	0.4 (32.3)	19 (64.4)	96.0	70.7
		*Total*	*49432*	*49439*	*1.5 (36.7)*	*12.9 (51.8)*	*94.0*	*79.6*
	WD	Natural	37646	35324	13 (43.0)	22 (54.3)	80.2	72.0
		Natural to Anthropogenic	4124	6448	4.1 (32.5)	21.6 (58.4)	91.0	71.1
		Anthropogenic	7662	7667	2.4 (33.6)	21.4 (60.5)	93.5	71.2
		*Total*	*49432*	*49439*	*10.6 (40.6)*	*21.8 (55.8)*	*83.2*	*71.7*
**South Coast**	WW	Natural	29864	27606	35.9 (48.1)	47.3 (60.4)	53.8	45.6
		Natural to Anthropogenic	5921	8179	82.6 (87.4)	88.6 (95.6)	12.3	8.3
		Anthropogenic	11527	11538	87.4 (90.8)	96.3 (98.0)	6.9	2.9
		*Total*	*47312*	*47323*	*54.3 (63.4)*	*66.4 (75.7)*	*37.2*	*28.7*
	WD	Natural	29864	27606	41.3 (53.3)	54.3 (65.4)	44.6	36.4
		Natural to Anthropogenic	5921	8179	80.7 (84.6)	89.3 (93.2)	12.4	7.4
		Anthropogenic	11527	11538	87.4 (89.4)	95.7 (97.5)	8.1	3.2
		*Total*	*47312*	*47323*	*57.4 (66.1)*	*70.4 (78.0)*	*31.7*	*23.3*

Climate scenarios (GCM) are abbreviated as WW (warmer wetter; CCCMA CGC 3.1) and WD (warmer drier; NCAR CCSM 3.0). Values in parentheses correspond to no dispersal scenarios, all other values correspond to unlimited dispersal scenarios. “Natural” land use corresponds to currently unconverted natural areas that remain unconverted under projected land use change. “Natural to anthropogenic” land use corresponds to currently unconverted areas that will be converted to anthropogenic uses under projected land use change. “Anthropogenic” land use corresponds to currently converted areas that will remain converted under projected land use change. The total area covered by each land use category is reported in Land area (km^2^).

After factoring in projected land use change, we find a disproportionate degree of spatial overlap between areas having losses in species richness due to climate change and areas that are either (1) already converted to anthropogenic land uses or (2) will be converted to anthropogenic land uses (Table 6). This pattern is most pronounced in the South Coast, where high rates of projected land use, particularly development, coincide with considerable habitat contraction driven by climate change for most of the CSS species considered in our study. Both already converted areas and natural areas with projected conversion to anthropogenic land uses in the South Coast have a significantly greater percentage of overlap with areas of projected species richness loss from climate change compared to natural areas in the South Coast without projected anthropogenic conversion (Table 6; all *P*<0.0001 after Bonferroni correction for multiple comparisons; Pearson's Chi-squared test). For example, assuming unlimited dispersal and a warmer wetter future climate in 2050, 82.6% of natural South Coast land with projected anthropogenic conversion (“natural to anthropogenic”) will undergo a loss in species richness due to projected climate change by 2080, compared to only 35.9% of South Coast natural lands without projected anthropogenic conversion (“natural”). We also find higher median losses in species richness due to climate change for areas with projected conversion to anthropogenic land uses compared to natural areas without projected conversion, a pattern that is consistent across time periods, climate scenarios, and dispersal scenarios (Figs. S2, S3).

For the most part, however, this pattern does not hold up for the Central Coast where projected species richness losses and gains due to climate change are similar across categories of projected anthropogenic land use change (Table 6, Figs. S2, S3). Only under a warmer wetter future climate in the 2080s (both dispersal scenarios), do we find a significantly greater percentage of overlap in projected species richness loss due to climate change and projected anthropogenic conversion compared to natural areas without projected anthropogenic conversion (all *P*<0.0001 after Bonferroni correction for multiple comparisons; Pearson's Chi-squared test). Thus, our results indicate that compounding impacts of projected land use and climate change on CSS will be centered primarily in the South Coast Ecoregion under the future scenarios considered in our study.

## Discussion

Given the current unprecedented rate of environmental change, successful conservation of biodiversity this century must address the potential impacts of both future land use and climate change on species and ecosystems. The disconnect between relatively high resolution downscaled climate projections and the coarse resolution treatment of land use-land cover change projections, however, has led to the exclusion of future land use from assessments of future environmental change impacts on species and ecosystems, which typically require local to regional scale analyses [9]. By using newly developed, downscaled scenario-based land use projections [9], [59], we were able to link future impacts of projected climate change and land use for CSS and examine multiple drivers of environmental change under a consistent future emission storyline (SRES A1B) at a regional scale and larger spatial extent than has previously been possible [34], [60].

Our analyses show that during the 21^st^ century, projected land use could pose a threat to CSS that is as large as that posed by projected climate change, if not larger. For many species, projected anthropogenic land use drove greater habitat losses compared to projected climate change, particularly during the first half of the century, which was consistent with the high rates of habitat conversion projected this century in both Central and South Coast Ecoregions. Interestingly, this pattern was only significant under assumptions of unlimited dispersal, where considerable gains in climatically suitable habitat offset some of the concurrent habitat losses from projected climate change. Projected land use within areas of climate-driven habitat gains, however, lowered potential habitat increases in scenarios of combined climate change and land use. We found an increase in the number of species where projected climate change drove greater habitat losses relative to land use during the second half of the century, suggesting that impacts from climate change may rise through the end of the century. Therefore, mitigating climate change impacts may become increasingly important for CSS management and conservation.

Overall, projected climate change impacts were highly variable across CSS species and heavily dependent on dispersal assumptions, highlighting the importance of dispersal in moderating habitat losses from both land use and climate and suggesting species responses to climate change may be highly individualized. The broad dispersal capacities of many CSS species with small, wind dispersed seeds (e.g., *A. californica, E. californica, E. fasciculatum, M. aurantiacus*) [46], could facilitate a northward expansion of CSS under projected climate change. Such range shifts, however, will also depend upon successful establishment and recruitment of CSS species within the mosaic of chaparral, CSS, and grasslands that currently dominates much of the central coast. These processes will ultimately be driven by both regional (climate) and local factors such as topography, geological substrate, disturbance, and species interactions [24], [43], [44], [46], [61]–[69].

For example, many wind-dispersed CSS species are able to invade areas of chaparral opened by disturbances such as fire [25], [65], [70]. However, short return intervals of fire, which will also be influenced by projected climate change [71] and urbanization [34], as well as high levels of other anthropogenic disturbances, facilitate conversion of shrublands to exotic grasslands [27], [46], [72]. Nitrogen deposition from pollution further reinforces this conversion [30], [73] and may impede the successful establishment of CSS in new, climatically suitable habitats under climate change. Furthermore, habitat fragmentation poses a formidable barrier to species migration, severely limiting the ability of a species to disperse across a landscape [74]. Thus, the future dynamics of CSS expansion will likely be complex, governed by many factors and processes that are also influenced by anthropogenic change.

Our findings also highlight the potential for future land use and climate change to have compounding negative impacts on CSS, particularly in the South Coast, where we find high geographic overlap in habitat losses driven by projected climate change and projected anthropogenic conversion. The rate of climate change and degree of habitat loss from land use both have thresholds beyond which the probability of population extinction becomes increasingly likely [75], [76]. As anthropogenic land use drives habitat loss to a threshold where local populations no longer have sufficient available habitat to persist, concurrent climate change may also surpass a critical rate at which population extinction becomes likely, the position of which may be lowered by habitat loss from land use [76]. Additionally, modeling studies indicate that habitat fragmentation and habitat quality may impact population extinction thresholds such that more habitat is required for population persistence in a fragmented landscape [77]. Fragmented landscapes may also have greater sensitivities to climate change [76].

California sage scrub's coastal distribution in lowland and relatively fertile areas with sizable human populations makes it particularly vulnerable to habitat conversion and fragmentation from development and agriculture [78]. Under a future of increasing land use and climate change, this sensitivity to human land use may result in the loss of CSS species that otherwise may have been able to keep pace with climate change without the additional pressures from land use, especially those species with low colonization ability and/or poor dispersal. Furthermore, our models likely underestimate the impact of projected land use, as we do not address habitat fragmentation, just total habitat loss.

As individual species shift in geographic distribution in response to climate change, patterns of richness and species assemblage could also change dramatically. Under the scenarios considered in our study, we see the potential for increasing richness along the Central Coast resulting from the northern habitat expansion of many CSS species. Though not examined in our study, this could result in novel, no-analog assemblages of species, where future communities and species interactions have no modern-day equivalent. Such community projections under 21^st^ century climate change have been shown for terrestrial breeding bird species in California [36]. In contrast, the South Coast could experience considerable declines in species richness from widespread southern habitat contraction under climate change, particularly in costal San Diego County, currently a region of high CSS floristic diversity and endemism [23]–[25], [29]. Both community reassembly and losses in richness have implications for the numerous sensitive species associated with CSS [31], [32].

Ultimately, the response of a species under climate change will be a function of the dynamics at both the leading (expanding) and trailing (low-latitude limit) edges of a species range [5], [79], which could have dramatically different processes and mechanisms. Within this context, conservation and management objectives may need to diverge between the Central Coast, where many species may gain climatically suitable habitat, and the South Coast where many species may lose climatically suitable habitat. If 21^st^ century climate change follows a similar trajectory as that examined in our study, the South Coast could represent the trailing edge of many CSS species ranges. The combined pressures of projected land use and climate change could severely impact local extinction at this trailing edge of CSS species ranges. Meanwhile habitat loss and fragmentation from projected land use in the leading edge of species ranges in the Central Coast could have a large impact on species migrations, severely limiting the ability of species to expand into habitat newly suitable under climate change.

Limiting future habitat conversion and fragmentation from land use is a strategy that could be broadly beneficial, both in preventing further barriers to dispersal so species are more likely to keep pace with climate change, and in maintaining patches of habitat above thresholds where climate change may drive local extinctions. In the South Coast, conservation efforts may need to prioritize the protection of remaining high quality habitat and maintenance of trailing edge populations, which could include developing action plans that mitigate future impacts from both drivers and promote species resilience to climate change. The future persistence of CSS may also hinge upon the successful establishment of species along the Central Coast; however, the role for managers is more complex in this case. How actively should managers facilitate species movements? Does the northern expansion of CSS come at the expense of local natives or other vegetation types? One approach that is likely to benefit a variety of species, no just CSS, is to promote species movements through the protection of strategic migration corridors.

We present a possible future trajectory of change for CSS and our results should be viewed as a hypothesis of how CSS may be impacted by projected land use and climate change. While we provide a direct comparison of two major drivers of future impact for CSS through our use of linked land use and climate change projections, we considered just one (SRES A1B) of many possible future storylines. Additionally, species distribution models are inherently uncertain, from the mechanisms driving species distributions, imperfect modeling methods, to the trajectories of future climate change and the extrapolation of species responses in novel future climates outside the range of contemporary climate used to parameterize models [80]. In using two GCMs, we show a range in the possible trajectory of projected climate change. Different GCMs and future emission scenarios may show different patterns of climate change severity and impact across California. Although uncertainties can also arise from SDM algorithms [81], [82], we chose a single algorithm, Maxent, rather than comparing multiple methods, as Maxent has high performance with the spatially biased, presence-only locality data used in our study [50]. Similarly, we use a single model and scenario of projected land use in California. While the land use projections used in our study are perhaps the most thematically and spatially detailed regional dataset available, they represent a first step in the development of tools and models [9], which will undoubtedly continue to be refined.

We were unable to compare or rank the uncertainty arising from each of our model components (climate projections, land use projections, modeling algorithm), which could potentially result in compounded uncertainty. Identifying the greatest sources of uncertainty within combined models is important to inform subsequent management decisions based upon model outputs. Sensitivity analysis may provide a method to compare sources of uncertainty in combined models and has been recently applied to coupled SDM and dynamic population models that incorporate combined impacts of environmental change (climate, land use, disturbance regimes) on species extinction risk [83].

The regional scale and 1 km spatial resolution of our analyses did not include microclimate variability that exists at finer spatial scales [35], which could result in over-predictions of current ranges and future habitat loss due to climate change. Steep microclimatic gradients, such as those due to rugged topography can facilitate species range shifts over shorter distances, making it more likely that a species could keep pace with changing climate. As our modeling framework did not address populations, which are typically dynamic and patchily distributed at local scales, our habitat maps also likely encompass geographically larger and more continuous areas than the current realized distribution of each species. In contrast, our estimates of land use impact are conservative, as we do not consider the additional effects of habitat fragmentation and degradation on CSS persistence and dispersal. Previous papers estimate anthropogenic activities have driven losses of up to 90% of CSS's original extent [25], [27]–[29], considerably higher than our median estimate of 35% current habitat conversion for individual shrub species.

Coupled models can improve predictions by combining dynamic population models and SDMs [84], [85], but require detailed species demographic data, which was not available for all 20 species we considered in our study. Additionally, our models do not incorporate dynamic processes that may buffer climate change, such as the capacity of a species for acclimation or adaptation to new environmental conditions. Nevertheless, our findings provide important insight and hypotheses into how 21^st^ century projected land use and climate change may impact CSS species and patterns of species richness. They can best be applied in combination with careful monitoring of CSS and climate change and land use impacts in an adaptive management context.

In conclusion, we emphasize the necessity to include analyses of both projected land use and climate change in conservation and resource management planning. We illustrate the potential for land use and climate change to have compounding negative impacts on CSS, particularly in southern California. We show the potential for the dynamics of CSS to diverge geographically under scenarios of future change, with strikingly different patterns of impact in the Central Coast, which may contain the expanding edge for many species ranges, and the South Coast, which may contain the trailing edge of many species ranges. The persistence and extent of CSS will likely hinge upon the protection of remaining critical habitat in southern California as well as the successful dispersal and establishment of species along the coastal central California. Thus, in the context of future environmental change, conservation objectives and management strategies may need to differ across species ranges and ecoregions.

## Supporting Information

Table S1 **Current (2000) and projected (2050, 2080) land cover and projected rate of land cover change (km^2^ yr^−1^) by California ecoregion.** Total anthropogenic land use is the sum of developed, cultivated cropland, hay/pasture, mining, and mechanically disturbed (logged) land uses. Current and projected land use-land cover data is from USGS LandCarbon [9].
(DOCX)

Table S2 **Projected conversion of current climatically suitable habitat of CSS species to anthropogenic land uses.** The area (km^2^) of currently unconverted suitable habitat (Unconvt.) and the percent of total currently suitable habitat already converted to anthropogenic land uses (Convt.) in 2000 are provided in the first two columns. Anthropogenic land uses are abbreviated as developed (D), cultivated crops (C), and hay/pasture (H/P). “Other” includes mechanically disturbed (logging) and mining. All habitat loss values are the percent change in unconverted current (2000) climatically suitable habitat. Projected land use-land cover data is from USGS LandCarbon [9].
(DOCX)

Table S3 **Percent change in CSS habitat due to projected land use and climate change under the warmer wetter (CCCMA CGC 3.1) scenario for 2000–2050, 2050–2080, and 2000–2080.** Abbreviations: climate change only scenario (CC only) and combined land use and climate change scenario (LU+CC).
(DOCX)

Table S4 **Percent change in CSS habitat due to projected land use and climate change under the warmer drier (NCAR CCSM 3.0) scenario for 2000–2050, 2050–2080, and 2000–2080.** Abbreviations: climate change only scenario (CC only) and combined land use and climate change scenario (LU+CC).
(DOCX)

Figure S1 **Projected Land use-land cover Maps.** USGS historical (2000) and projected (2050, 2080) land use-land cover maps in Central Coast and South Coast Ecoregions of California (back outlines). Projected land use-land cover corresponds to the IPCC-SRES A1B future scenario. All land cover data was resampled to 1 km resolution from the USGS LandCarbon 250 m resolution land-cover-land use maps for the continental United States [9].
(TIF)

Figure S2 **Distribution of projected change in CSS richness due to climate change for different land use categories (Fig S2: unlimited dispersal, Fig S3: no dispersal).** Figure panels show the distribution (percent of ecoregion land area) of projected change in CSS species richness due to climate change assuming unlimited dispersal (S2) and no dispersal (S3). The top row shows the distribution of projected CSS richness change for the entire ecoregion (Central Coast or South Coast). The next three rows show projected CSS richness change for three categories of projected land use (Natural, Natural to Anthropogenic, and Anthropogenic) within each ecoregion. “Natural” land use corresponds to currently unconverted natural areas that will remain unconverted under projected land use change. “Natural to anthropogenic” land use corresponds to currently unconverted areas that will be converted to anthropogenic uses under projected land use change. “Anthropogenic” land use corresponds to currently converted areas that will remain converted under projected land use change. Gray lines represent early century (2050s) modeled species richness and black lines represent late century (2080s) modeled species richness. The dashed line indicates zero change in species richness.
(TIF)

Figure S3 **Distribution of projected change in CSS richness due to climate change for different land use categories (Fig S2: unlimited dispersal, Fig S3: no dispersal).** Figure panels show the distribution (percent of ecoregion land area) of projected change in CSS species richness due to climate change assuming unlimited dispersal (S2) and no dispersal (S3). The top row shows the distribution of projected CSS richness change for the entire ecoregion (Central Coast or South Coast). The next three rows show projected CSS richness change for three categories of projected land use (Natural, Natural to Anthropogenic, and Anthropogenic) within each ecoregion. “Natural” land use corresponds to currently unconverted natural areas that will remain unconverted under projected land use change. “Natural to anthropogenic” land use corresponds to currently unconverted areas that will be converted to anthropogenic uses under projected land use change. “Anthropogenic” land use corresponds to currently converted areas that will remain converted under projected land use change. Gray lines represent early century (2050s) modeled species richness and black lines represent late century (2080s) modeled species richness. The dashed line indicates zero change in species richness.
(TIF)
