# Supplementary material for: Land Use Compounds Habitat Losses under Projected Climate Change in a Threatened California Ecosystem
Source: PLoS One. 2014 Jan 21;9(1):e86487. doi: 10.1371/journal.pone.0086487 (PMC3897708; doi:10.1371/journal.pone.0086487)
Supplement: Table S1 — Current (2000) and projected (2050, 2080) land cover and projected rate of land cover change (km2 yr−1) by California ecoregion. Total anthropogenic land use is the sum of developed, cultivated cropland, hay/pasture, mining, and mechanically disturbed (logged) land uses. Current and projected land use-land cover data is from USGS LandCarbon [9]. (DOCX) [file pone.0086487.s001.docx]

**Table S1** Current (2000) and projected (2050, 2080) land cover and projected rate of land cover change (km^2^ yr^-1^) by California ecoregion. Total anthropogenic land use is the sum of developed, cultivated cropland, hay/pasture, mining, and mechanically disturbed (logged) land uses. Current and projected land use-land cover data is from USGS LandCarbon [[9](#_ENREF_9)].

| **Land cover** | **2000** | | **2050** | | **2080** | | **Rate of Change (km^2^ yr^-1^)** | |
| --- | --- | --- | --- | --- | --- | --- | --- | --- |
|  | **Area (km^2^)** | **%** | **Area (km^2^)** | **%** | **Area (km^2^)** | **%** | **2000– 2050** | **2050– 2080** |
| **Central Coast** | 49574 |  |  |  |  |  |  |  |
| Grassland | 18843 | 38.0 | 15735 | 31.7 | 14020 | 28.3 | -62 | -57 |
| Shrubland | 8891 | 17.9 | 8128 | 16.4 | 7544 | 15.2 | -15 | -19 |
| Evergreen Forest | 8340 | 16.8 | 8178 | 16.5 | 8146 | 16.4 | -3 | -1 |
| Mixed Forest | 2918 | 5.9 | 2929 | 5.9 | 2930 | 5.9 | < 1 | < 1 |
| Deciduous Forest | 1274 | 2.6 | 1266 | 2.6 | 1265 | 2.6 | < 1 | < 1 |
| Barren | 1146 | 2.3 | 1167 | 2.4 | 1167 | 2.4 | < 1 | 0 |
| Wetland | 50 | 0.1 | 31 | 0.1 | 29 | 0.1 | < 1 | < 1 |
| Water | 361 | 0.7 | 368 | 0.7 | 366 | 0.7 | < 1 | < 1 |
| Developed | 3548 | 7.2 | 6956 | 14.0 | 8209 | 16.6 | 68 | 42 |
| Cultivated Cropland | 2446 | 4.9 | 2997 | 6.0 | 3859 | 7.8 | 11 | 29 |
| Hay/Pasture | 1681 | 3.4 | 1678 | 3.4 | 1913 | 3.9 | < 1 | 8 |
| Mechanically disturbed | 30 | 0.1 | 92 | 0.2 | 78 | 0.2 | 1 | < 1 |
| Mining | 46 | 0.1 | 49 | 0.1 | 48 | 0.1 | < 1 | < 1 |
| Total Anthropogenic | 7751 | 15.6 | 11772 | 23.7 | 14107 | 28.5 | 80 | 78 |
| **South Coast** | 47432 |  |  |  |  |  |  |  |
| Grassland | 5508 | 11.6 | 3730 | 7.9 | 3309 | 7.0 | -36 | -14 |
| Shrubland | 21468 | 45.3 | 17584 | 37.1 | 15810 | 33.3 | -78 | -59 |
| Evergreen Forest | 6019 | 12.7 | 5929 | 12.5 | 5885 | 12.4 | -2 | -1 |
| Mixed Forest | 1295 | 2.7 | 1267 | 2.7 | 1248 | 2.6 | -1 | -1 |
| Deciduous Forest | 478 | 1.0 | 468 | 1.0 | 464 | 1.0 | < 1 | < 1 |
| Barren | 825 | 1.7 | 798 | 1.7 | 798 | 1.7 | -1 | 0 |
| Wetland | 50 | 0.1 | 35 | 0.1 | 35 | 0.1 | < 1 | 0 |
| Water | 261 | 0.6 | 258 | 0.5 | 255 | 0.5 | < 1 | < 1 |
| Developed | 8869 | 18.7 | 15161 | 32.0 | 17248 | 36.4 | 126 | 70 |
| Cultivated Cropland | 1865 | 3.9 | 1674 | 3.5 | 1896 | 4.0 | -4 | 7 |
| Hay/Pasture | 768 | 1.6 | 513 | 1.1 | 472 | 1.0 | -5 | -1 |
| Mechanically disturbed | 12 | < 0.1 | 5 | < 0.1 | 2 | < 0.1 | < 1 | < 1 |
| Mining | 14 | < 0.1 | 10 | < 0.1 | 10 | < 0.1 | < 1 | 0 |
| Total Anthropogenic | 11528 | 24.3 | 17363 | 36.6 | 19628 | 41.4 | 117 | 76 |
