# Supplementary material for: Land Use Compounds Habitat Losses under Projected Climate Change in a Threatened California Ecosystem
Source: PLoS One. 2014 Jan 21;9(1):e86487. doi: 10.1371/journal.pone.0086487 (PMC3897708; doi:10.1371/journal.pone.0086487)
Supplement: Table S2 — Projected conversion of current climatically suitable habitat of CSS species to anthropogenic land uses. The area (km2) of currently unconverted suitable habitat (Unconvt.) and the percent of total currently suitable habitat already converted to anthropogenic land uses (Convt.) in 2000 are provided in the first two columns. Anthropogenic land uses are abbreviated as developed (D), cultivated crops (C), and hay/pasture (H/P). “Other” includes mechanically disturbed (logging) and mining. All habitat loss values are the percent change in unconverted current (2000) climatically suitable habitat. Projected land use-land cover data is from USGS LandCarbon [9]. (DOCX) [file pone.0086487.s002.docx]

**Table S2** Projected conversion of current climatically suitable habitat of CSS species to anthropogenic land uses. The area (km^2^) of currently unconverted suitable habitat (Unconvt.) and the percent of total currently suitable habitat already converted to anthropogenic land uses (Convt.) in 2000 are provided in the first two columns. Anthropogenic land uses are abbreviated as developed (D), cultivated crops (C), and hay/pasture (H/P). “Other” includes mechanically disturbed (logging) and mining. All habitat loss values are the percent change in unconverted current (2000) climatically suitable habitat. Projected land use-land cover data is from USGS LandCarbon [[9](#_ENREF_9)].

| Species | Current suitable habitat | | Percent loss in habitat to anthropogenic land use | | | | | | | | | | | | | | | | | |
| --- | --- | --- | --- | --- | --- | --- | --- | --- | --- | --- | --- | --- | --- | --- | --- | --- | --- | --- | --- | --- |
|  |  |  | 2000–2050 | | | | | | 2050–2080 | | | | | | 2000–2080 | | | | | |
|  | Unconvt. (km^2^) | Convt. (%) | | D | C | H/P | Other | Total | | D | C | H/P | Other | Total | | D | C | H/P | Other | Total |
| *Acmispon glaber* | 70717 | 27.5 | | 12.7 | 3.0 | 1.2 | 0.1 | 17.0 | | 4.2 | 2.1 | 0.9 | 0.1 | 7.4 | | 17.3 | 4.8 | 1.9 | 0.1 | 24.1 |
| *Artemisia californica* | 35106 | 33.2 | | 21.3 | 2.8 | 1.1 | 0.1 | 25.3 | | 6.2 | 2.1 | 0.6 | < 0.1 | 9.0 | | 28.1 | 4.4 | 1.5 | 0.1 | 34.1 |
| *Bahiopsis laciniata* | 18144 | 44.4 | | 27.1 | 4.7 | 1.6 | < 0.1 | 33.4 | | 6.7 | 3.5 | 0.8 | < 0.1 | 11.0 | | 34.6 | 7.7 | 2.0 | < 0.1 | 44.3 |
| *Cneoridium dumosum* | 11815 | 47.5 | | 26.6 | 4.0 | 1.4 | < 0.1 | 31.9 | | 6.3 | 2.9 | 0.7 | 0.0 | 9.8 | | 33.7 | 6.4 | 1.7 | 0.0 | 41.7 |
| *Encelia californica* | 21313 | 42.8 | | 28.1 | 3.7 | 1.2 | < 0.1 | 33.1 | | 7.6 | 2.5 | 0.7 | < 0.1 | 10.9 | | 36.6 | 5.6 | 1.6 | < 0.1 | 43.8 |
| *Ericameria ericoides* | 11862 | 38.5 | | 15.9 | 2.8 | 1.8 | < 0.1 | 21.1 | | 6.1 | 2.2 | 1.1 | 0.3 | 9.7 | | 22.6 | 4.7 | 2.6 | 0.3 | 30.2 |
| *Eriogonum fasciculatum* | 75088 | 24.8 | | 10.8 | 2.8 | 1.1 | 0.1 | 14.9 | | 3.3 | 2.2 | 0.9 | 0.1 | 6.5 | | 14.4 | 4.7 | 1.8 | 0.1 | 21.1 |
| *Hazardia squarrosa* | 68557 | 27.2 | | 11.9 | 2.9 | 1.2 | 0.1 | 16.1 | | 3.9 | 2.2 | 0.9 | < 0.1 | 7.0 | | 16.1 | 4.8 | 1.9 | 0.1 | 22.8 |
| *Hesperoyucca whipplei* | 70234 | 19.4 | | 9.7 | 2.0 | 0.9 | 0.1 | 12.7 | | 3.3 | 1.7 | 0.8 | 0.1 | 5.9 | | 13.2 | 3.5 | 1.5 | 0.1 | 18.3 |
| *Isocoma menziesii* | 36897 | 37.3 | | 18.3 | 4.4 | 1.8 | < 0.1 | 24.6 | | 5.5 | 3.1 | 1.2 | < 0.1 | 9.9 | | 24.4 | 7.0 | 2.6 | < 0.1 | 34.1 |
| *Malosma laurina* | 26074 | 38.6 | | 24.3 | 3.3 | 1.3 | < 0.1 | 28.9 | | 6.7 | 2.5 | 0.8 | < 0.1 | 9.9 | | 31.6 | 5.3 | 1.7 | < 0.1 | 38.7 |
| *Mimulus aurantiacus* | 113938 | 22.3 | | 8.9 | 2.4 | 1.2 | 0.6 | 13.1 | | 3.3 | 1.7 | 0.9 | 0.7 | 6.6 | | 12.4 | 3.8 | 2.0 | 0.8 | 19.0 |
| *Mirabilis laevis var. crassifolia* | 48134 | 36.1 | | 17.1 | 4.3 | 1.7 | 0.1 | 23.1 | | 5.1 | 3.0 | 1.0 | < 0.1 | 9.2 | | 22.7 | 6.9 | 2.4 | 0.1 | 32.1 |
| *Opuntia littoralis* | 16915 | 44.5 | | 30.4 | 3.5 | 0.9 | < 0.1 | 34.8 | | 8.1 | 2.4 | 0.4 | < 0.1 | 10.9 | | 39.5 | 5.1 | 1.0 | < 0.1 | 45.6 |
| *Rhus integrifolia* | 17877 | 42.7 | | 28.0 | 3.2 | 0.9 | < 0.1 | 32.2 | | 7.5 | 2.1 | 0.6 | < 0.1 | 10.2 | | 36.3 | 4.7 | 1.2 | < 0.1 | 42.2 |
| *Ribes speciosum* | 44160 | 28.7 | | 14.9 | 2.1 | 1.3 | 0.3 | 18.6 | | 4.9 | 1.6 | 0.9 | 0.2 | 7.6 | | 20.2 | 3.5 | 1.9 | 0.2 | 25.8 |
| *Salvia apiana* | 31515 | 28.9 | | 16.2 | 2.0 | 0.4 | < 0.1 | 18.7 | | 4.9 | 1.8 | 0.3 | < 0.1 | 7.0 | | 21.5 | 3.4 | 0.6 | < 0.1 | 25.5 |
| *Salvia leucophylla* | 78252 | 29.6 | | 11.4 | 3.2 | 1.4 | 0.1 | 16.0 | | 4.0 | 2.1 | 1.1 | 0.1 | 7.3 | | 15.6 | 5.0 | 2.3 | 0.1 | 23.0 |
| *Salvia mellifera* | 50545 | 31.7 | | 16.3 | 3.2 | 1.2 | 0.1 | 20.8 | | 5.0 | 2.1 | 0.8 | 0.1 | 8.0 | | 21.7 | 4.9 | 1.8 | 0.1 | 28.5 |
| *Xylococcus bicolor* | 17248 | 40.7 | | 25.4 | 3.3 | 1.4 | < 0.1 | 30.1 | | 6.4 | 2.8 | 0.9 | < 0.1 | 10.0 | | 32.4 | 5.6 | 2.0 | < 0.1 | 40.0 |
