# Supplementary material for: Land Use Compounds Habitat Losses under Projected Climate Change in a Threatened California Ecosystem
Source: PLoS One. 2014 Jan 21;9(1):e86487. doi: 10.1371/journal.pone.0086487 (PMC3897708; doi:10.1371/journal.pone.0086487)
Supplement: Table S3 — Percent change in CSS habitat due to projected land use and climate change under the warmer wetter (CCCMA CGC 3.1) scenario for 2000–2050, 2050–2080, and 2000–2080. Abbreviations: climate change only scenario (CC only) and combined land use and climate change scenario (LU+CC). (DOCX) [file pone.0086487.s003.docx]

**Table S3** Percent change in CSS habitat due to projected land use and climate change under the warmer wetter (CCCMA CGC 3.1) scenario for 2000–2050, 2050–2080, and 2000–2080. Abbreviations: climate change only scenario (CC only) and combined land use and climate change scenario (LU + CC).

| Species | Percent change in habitat | | | | | | | | | | | |
| --- | --- | --- | --- | --- | --- | --- | --- | --- | --- | --- | --- | --- |
|  | CC only (no dispersal) | | | CC + LU (no dispersal) | | | CC only (unlimited dispersal) | | | CC + LU (unlimited dispersal) | | |
|  | 2000-2050 | 2050-2080 | 2000-2080 | 2000-2050 | 2050-2080 | 2000-2080 | 2000-2050 | 2050-2080 | 2000-2080 | 2000-2050 | 2050-2080 | 2000-2080 |
| *Acmispon glaber* | -7.2 | -3.1 | -8.5 | -23.0 | -9.6 | -30.2 | 34.4 | 17.2 | 52.4 | 15.4 | 6.6 | 22.4 |
| *Artemisia californica* | -4.3 | -3.8 | -9.6 | -28.4 | -12.1 | -40.1 | 73.3 | 18.6 | 89.5 | 43.7 | 5.8 | 49.9 |
| *Bahiopsis laciniata* | -19.0 | -14.6 | -41.2 | -45.9 | -20.9 | -66.4 | 69.5 | -26.1 | 33.5 | 30.1 | -37.5 | -7.0 |
| *Cneoridium dumosum* | -31.6 | -10.3 | -45.5 | -53.1 | -15.4 | -66.6 | -17.4 | -9.4 | -33.0 | -43.6 | -16.2 | -59.5 |
| *Encelia californica* | -7.3 | -3.1 | -9.0 | -37.4 | -12.3 | -48.0 | 118.8 | 12.8 | 129.3 | 74.5 | -5.4 | 70.4 |
| *Ericameria ericoides* | -12.3 | -6.8 | -20.8 | -29.6 | -14.8 | -44.0 | 9.5 | -11.4 | -4.0 | -9.3 | -20.6 | -29.2 |
| *Eriogonum fasciculatum* | -27.1 | -18.2 | -49.2 | -37.3 | -21.0 | -57.7 | -8.3 | -14.0 | -26.7 | -19.6 | -17.5 | -36.9 |
| *Hazardia squarrosa* | -24.2 | -13.9 | -40.9 | -35.2 | -17.4 | -51.8 | -15.7 | -9.7 | -28.8 | -26.9 | -13.3 | -40.0 |
| *Hesperoyucca whipplei* | -19.8 | -9.6 | -31.8 | -28.7 | -13.2 | -41.5 | 9.0 | -10.5 | -5.1 | -2.9 | -15.5 | -18.1 |
| *Isocoma menziesii* | -10.7 | -5.1 | -16.6 | -33.2 | -13.5 | -46.2 | 6.7 | -4.4 | 1.2 | -17.5 | -13.8 | -31.2 |
| *Malosma laurina* | -13.3 | -6.5 | -22.6 | -38.6 | -14.2 | -52.7 | 54.5 | 11.5 | 64.1 | 22.1 | -1.5 | 21.2 |
| *Mimulus aurantiacus* | -30.4 | -15.6 | -39.4 | -37.7 | -18.3 | -46.8 | 17.0 | 1.1 | 15.4 | 8.1 | -4.1 | 5.4 |
| *Mirabilis laevis var. crassifolia* | -22.6 | -15.6 | -44.3 | -39.7 | -20.5 | -59.3 | 48.4 | 9.5 | 50.6 | 25.8 | 1.1 | 27.5 |
| *Opuntia littoralis* | -0.2 | -0.2 | -0.5 | -35.0 | -11.0 | -45.8 | 243.9 | 45.6 | 288.3 | 176.9 | 15.7 | 193.9 |
| *Rhus integrifolia* | -3.1 | -4.0 | -11.5 | -33.8 | -13.0 | -46.6 | 135.2 | 44.5 | 178.2 | 84.8 | 21.8 | 108.1 |
| *Ribes speciosum* | -12.2 | -9.6 | -22.0 | -28.2 | -14.7 | -39.8 | 1.7 | -1.1 | -1.5 | -15.1 | -7.7 | -22.2 |
| *Salvia apiana* | -40.4 | -20.2 | -62.6 | -45.5 | -21.7 | -66.3 | 79.8 | -28.4 | 44.5 | 62.6 | -34.7 | 28.4 |
| *Salvia leucophylla* | -18.8 | -6.8 | -26.6 | -29.9 | -11.4 | -41.0 | 16.2 | -0.1 | 15.2 | 3.2 | -6.3 | -2.7 |
| *Salvia mellifera* | -10.6 | -9.0 | -22.1 | -28.4 | -15.2 | -43.1 | 41.1 | 6.8 | 45.7 | 20.2 | -3.2 | 17.7 |
| *Xylococcus bicolor* | -50.2 | -24.3 | -83.0 | -64.6 | -25.4 | -89.9 | -15.1 | -24.5 | -50.0 | -35.9 | -27.3 | -62.8 |
