# Supplementary material for: Land Use Compounds Habitat Losses under Projected Climate Change in a Threatened California Ecosystem
Source: PLoS One. 2014 Jan 21;9(1):e86487. doi: 10.1371/journal.pone.0086487 (PMC3897708; doi:10.1371/journal.pone.0086487)
Supplement: Table S4 — Percent change in CSS habitat due to projected land use and climate change under the warmer drier (NCAR CCSM 3.0) scenario for 2000–2050, 2050–2080, and 2000–2080. Abbreviations: climate change only scenario (CC only) and combined land use and climate change scenario (LU+CC). (DOCX) [file pone.0086487.s004.docx]

**Table S4** Percent change in CSS habitat due to projected land use and climate change under the warmer drier (NCAR CCSM 3.0) scenario for 2000–2050, 2050–2080, and 2000–2080. Abbreviations: climate change only scenario (CC only) and combined land use and climate change scenario (LU + CC).

| Species | Percent change in habitat | | | | | | | | | | | |
| --- | --- | --- | --- | --- | --- | --- | --- | --- | --- | --- | --- | --- |
|  | CC only (no dispersal) | | | CC + LU (no dispersal) | | | CC only (unlimited dispersal) | | | CC + LU (unlimited dispersal) | | |
|  | 2000-2050 | 2050-2080 | 2000-2080 | 2000-2050 | 2050-2080 | 2000-2080 | 2000-2050 | 2050-2080 | 2000-2080 | 2000-2050 | 2050-2080 | 2000-2080 |
| *Acmispon glaber* | -11.8 | -4.5 | -16.5 | -26.7 | -10.2 | -35.3 | 19.9 | 1.3 | 19.7 | 3.4 | -6.2 | -2.5 |
| *Artemisia californica* | -9.9 | -5.5 | -15.5 | -33.8 | -13.4 | -46.1 | 36.3 | 15.3 | 50.7 | 9.1 | 4.7 | 14.1 |
| *Bahiopsis laciniata* | -11.4 | -8.7 | -23.4 | -40.5 | -16.7 | -56.9 | 97.7 | 12.2 | 106.5 | 53.7 | -4.2 | 50.2 |
| *Cneoridium dumosum* | -15.3 | -6.4 | -23.1 | -41.6 | -13.6 | -53.5 | 52.8 | -8.0 | 41.1 | 13.4 | -20.4 | -6.8 |
| *Encelia californica* | -9.1 | -2.9 | -10.8 | -38.5 | -12.1 | -49.8 | 125.0 | 39.6 | 164.4 | 80.6 | 19.0 | 101.0 |
| *Ericameria ericoides* | -8.5 | -3.7 | -13.0 | -26.3 | -12.4 | -37.7 | 32.7 | 5.3 | 36.6 | 10.5 | -7.2 | 5.1 |
| *Eriogonum fasciculatum* | -26.1 | -20.3 | -50.5 | -37.0 | -23.1 | -59.4 | -10.0 | -17.5 | -32.0 | -22.2 | -21.2 | -43.1 |
| *Hazardia squarrosa* | -23.7 | -16.7 | -43.7 | -35.5 | -20.3 | -55.3 | -13.3 | -14.0 | -30.8 | -25.4 | -17.7 | -43.0 |
| *Hesperoyucca whipplei* | -32.3 | -14.0 | -48.0 | -40.3 | -16.7 | -56.1 | -12.3 | -12.9 | -28.0 | -22.1 | -16.6 | -38.5 |
| *Isocoma menziesii* | -8.2 | -2.2 | -10.5 | -31.2 | -11.2 | -42.1 | 26.5 | 7.2 | 34.2 | 0.3 | -4.1 | -3.6 |
| *Malosma laurina* | -10.0 | -4.6 | -16.8 | -36.2 | -12.9 | -49.0 | 72.2 | 14.3 | 85.1 | 38.4 | 0.0 | 39.3 |
| *Mimulus aurantiacus* | -43.6 | -18.1 | -61.2 | -50.0 | -20.3 | -67.3 | -6.8 | -10.0 | -18.8 | -14.4 | -14.1 | -27.2 |
| *Mirabilis laevis var. crassifolia* | -20.2 | -11.4 | -36.3 | -39.0 | -17.3 | -55.7 | 72.2 | -0.1 | 65.5 | 46.7 | -9.3 | 38.1 |
| *Opuntia littoralis* | -3.6 | -2.0 | -6.8 | -36.9 | -12.0 | -48.8 | 192.4 | 48.6 | 240.1 | 130.0 | 20.9 | 151.9 |
| *Rhus integrifolia* | -10.7 | -8.4 | -22.8 | -38.9 | -15.7 | -54.4 | 147.9 | 45.8 | 190.3 | 98.3 | 23.3 | 123.6 |
| *Ribes speciosum* | -17.4 | -11.8 | -31.2 | -32.9 | -16.2 | -46.8 | 2.6 | -6.7 | -8.2 | -14.3 | -13.1 | -26.4 |
| *Salvia apiana* | -34.5 | -20.2 | -55.9 | -41.7 | -21.9 | -61.9 | 48.5 | -21.1 | 21.3 | 32.9 | -26.1 | 7.0 |
| *Salvia leucophylla* | -33.4 | -16.7 | -53.4 | -41.9 | -19.4 | -61.2 | -15.3 | -14.0 | -32.6 | -24.3 | -17.3 | -41.3 |
| *Salvia mellifera* | -13.4 | -9.0 | -25.0 | -30.7 | -15.0 | -45.5 | 25.1 | -4.8 | 17.5 | 5.2 | -12.7 | -7.2 |
| *Xylococcus bicolor* | -32.5 | -20.8 | -59.9 | -51.4 | -24.2 | -75.4 | 55.5 | -0.3 | 47.6 | 23.8 | -9.5 | 14.9 |
