# Supplementary figures and images for: Land Use Compounds Habitat Losses under Projected Climate Change in a Threatened California Ecosystem
Source: PLoS One. 2014 Jan 21;9(1):e86487. doi: 10.1371/journal.pone.0086487 (PMC3897708; doi:10.1371/journal.pone.0086487)

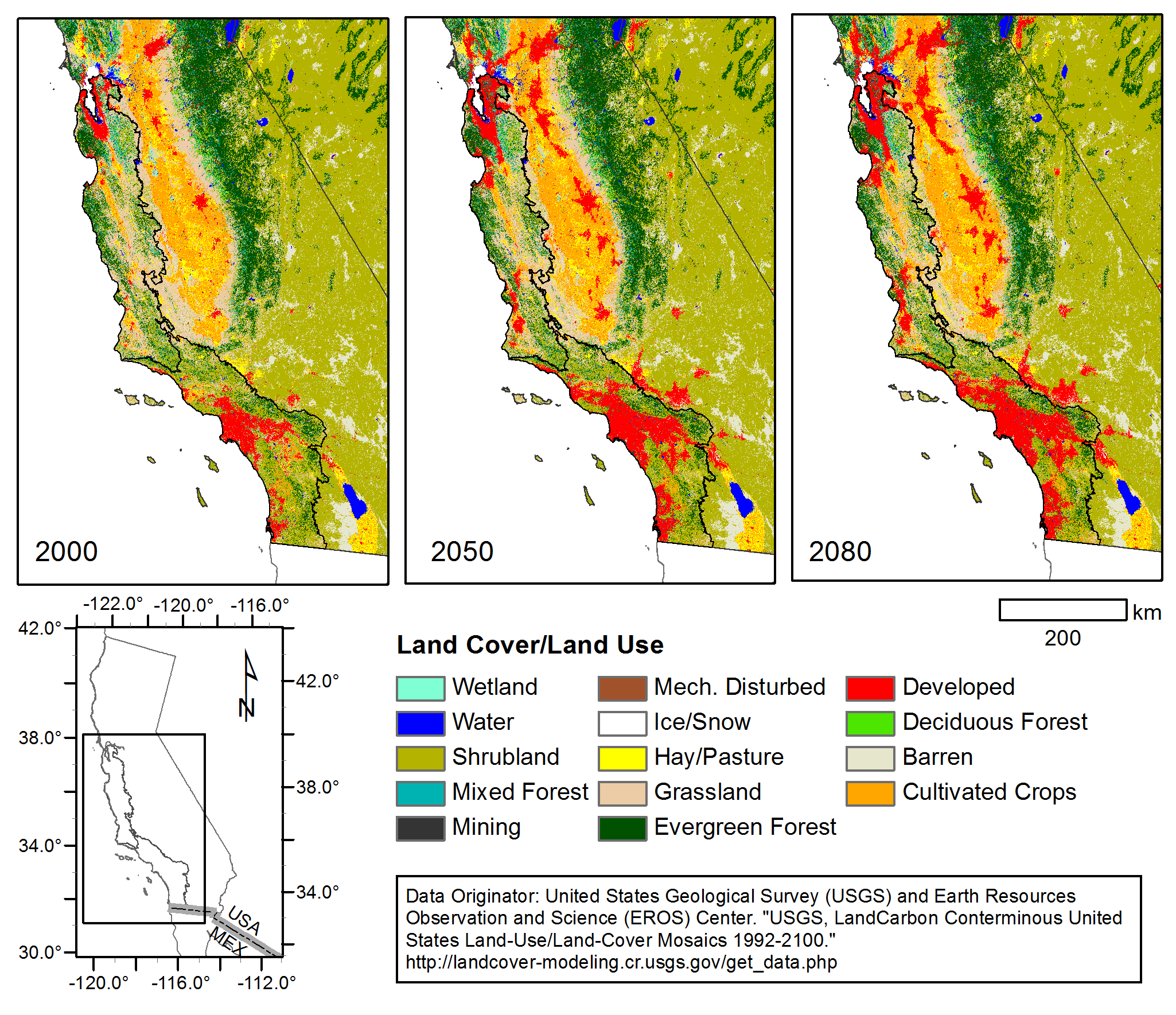

Supplement: Figure S1 — Projected Land use-land cover Maps. USGS historical (2000) and projected (2050, 2080) land use-land cover maps in Central Coast and South Coast Ecoregions of California (back outlines). Projected land use-land cover corresponds to the IPCC-SRES A1B future scenario. All land cover data was resampled to 1 km resolution from the USGS LandCarbon 250 m resolution land-cover-land use maps for the continental United States [9]. (TIF) [file pone.0086487.s005.tif]

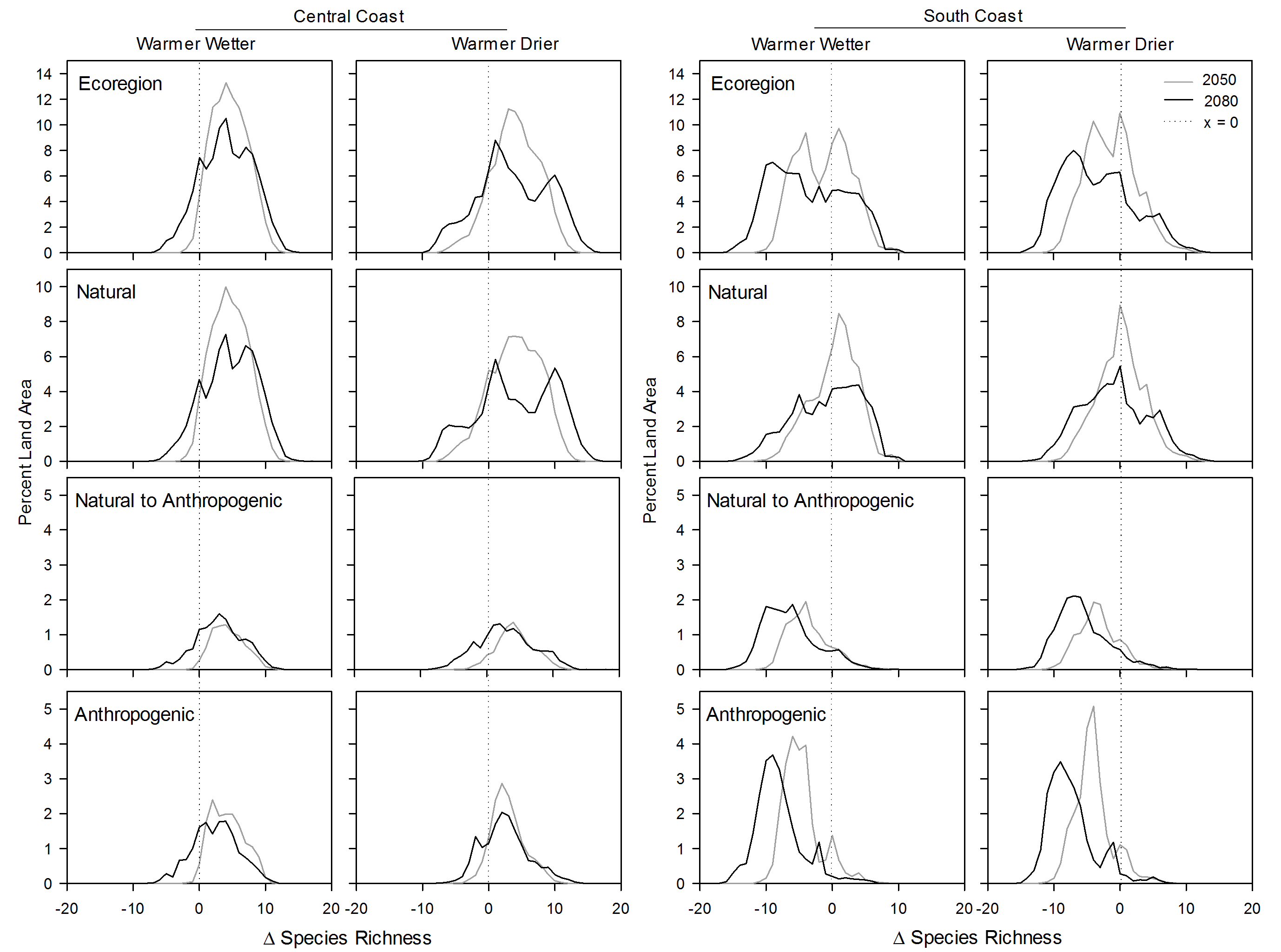

Supplement: Figure S2 — Distribution of projected change in CSS richness due to climate change for different land use categories (Fig S2: unlimited dispersal, Fig S3: no dispersal). Figure panels show the distribution (percent of ecoregion land area) of projected change in CSS species richness due to climate change assuming unlimited dispersal (S2) and no dispersal (S3). The top row shows the distribution of projected CSS richness change for the entire ecoregion (Central Coast or South Coast). The next three rows show projected CSS richness change for three categories of projected land use (Natural, Natural to Anthropogenic, and Anthropogenic) within each ecoregion. “Natural” land use corresponds to currently unconverted natural areas that will remain unconverted under projected land use change. “Natural to anthropogenic” land use corresponds to currently unconverted areas that will be converted to anthropogenic uses under projected land use change. “Anthropogenic” land use corresponds to currently converted areas that will remain converted under projected land use change. Gray lines represent early century (2050s) modeled species richness and black lines represent late century (2080s) modeled species richness. The dashed line indicates zero change in species richness. (TIF) [file pone.0086487.s006.tif]

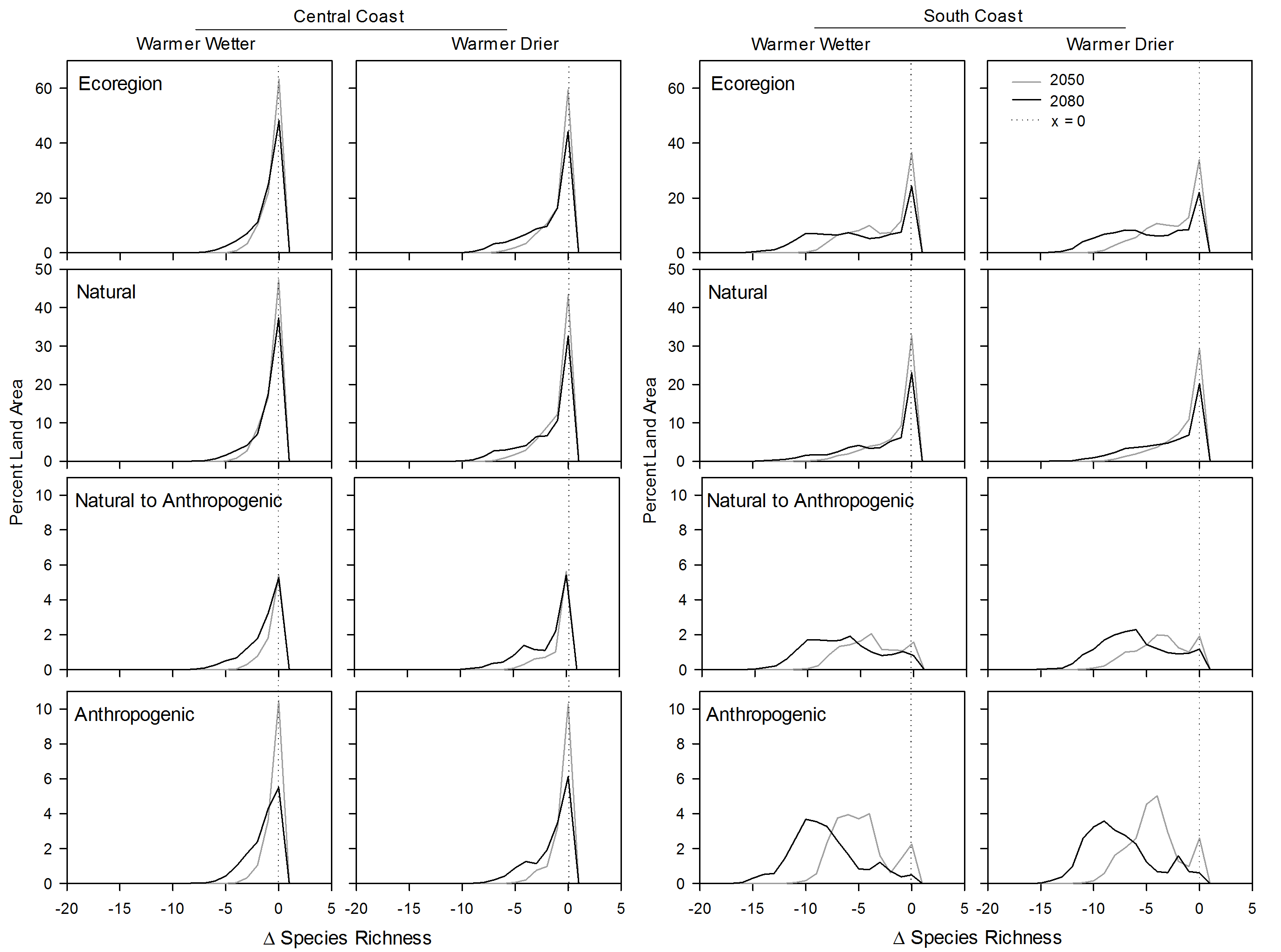

Supplement: Figure S3 — Distribution of projected change in CSS richness due to climate change for different land use categories (Fig S2: unlimited dispersal, Fig S3: no dispersal). Figure panels show the distribution (percent of ecoregion land area) of projected change in CSS species richness due to climate change assuming unlimited dispersal (S2) and no dispersal (S3). The top row shows the distribution of projected CSS richness change for the entire ecoregion (Central Coast or South Coast). The next three rows show projected CSS richness change for three categories of projected land use (Natural, Natural to Anthropogenic, and Anthropogenic) within each ecoregion. “Natural” land use corresponds to currently unconverted natural areas that will remain unconverted under projected land use change. “Natural to anthropogenic” land use corresponds to currently unconverted areas that will be converted to anthropogenic uses under projected land use change. “Anthropogenic” land use corresponds to currently converted areas that will remain converted under projected land use change. Gray lines represent early century (2050s) modeled species richness and black lines represent late century (2080s) modeled species richness. The dashed line indicates zero change in species richness. (TIF) [file pone.0086487.s007.tif]
